# Computational modelling identifies primary mediators of crosstalk between DNA damage and oxidative stress responses

**DOI:** 10.1371/journal.pcbi.1012844

**Published:** 2025-03-10

**Authors:** Elsje J. Burgers, Raju P. Sharma, Carl Joshua S. Eugenio, Muriel M. Heldring, Lukas S. Wijaya, Bob van de Water, Joost B. Beltman

**Affiliations:** 1 Division of Cell Systems and Drug Safety, Leiden Academic Centre for Drug Research, Leiden University, Leiden, The Netherlands; Georgia Institute of Technology and Emory University,UNITED STATES OF AMERICA

## Abstract

Cells exposed to toxicants, such as drugs, activate a wide variety of stress pathways, often simultaneously. Two important pathways that can influence cell fate and consequently adverse reactions are the oxidative stress response (OSR) and the DNA damage response (DDR). Previous studies have presented evidence of crosstalk between the OSR and DDR. We aimed to develop computational models to describe experimentally observed dynamics of both OSR and DDR proteins in liver (HepG2) cells *in vitro* upon exposure to various concentrations of either diethyl maleate (DEM; an agent primarily invoking oxidative stress) or etoposide (an agent primarily causing DNA damage). With these models, we aimed to identify the key interactions that cause crosstalk and their importance in describing protein dynamics. We developed a new model for the OSR pathway, coupled it to a previously developed model for the DDR pathway, and extended the resulting combined model based on multiple potential modes of crosstalk described in the literature. The different models were applied to previously published data of HepG2 GFP-reporter cells with time-dynamic information on the relative amount of proteins important for the OSR (NRF2, SRXN1) or DDR (p53, p21, BTG2 and MDM2). The developed models properly described key OSR and DDR protein dynamics, and *in silico* knockdowns of key model components in most cases led to a moderate effect on the connected pathway. The largest effect occurred after knockdown of p21, which resulted in a substantial decrease in NRF2 and SRXN1. We expect these models could play a role in adversity predictions by coupling our models with other models that predict cell fate or adversity based on the expression of specific proteins.

## Author summary

When cells are exposed to toxic substances, they can activate various pathways to regain homeostasis. Two important pathways include the oxidative stress response and the DNA damage response, which are also known to interact. In this study, we developed mathematical models combining the two pathways and including relevant interactions. We exploited data on key proteins for each pathway, acquired in cells originating from liver. The cells were exposed to etoposide (a DNA damage-inducing compound) or diethyl maleate (an oxidative stress-inducing compound). By calibrating multiple model variants to the data, we identified the key interactions between the two pathways. In the future, our models could also contribute to improved toxicity predictions, because both pathways have a role in potential adversity of chemicals or drugs.

## Introduction

Drug-Induced Liver Injury (DILI) is a frequent adverse drug reaction and a major problem for the pharmaceutical industry. Hepatic toxicity is the main reason both for why drugs fail during clinical trials [1] and for why drugs are withdrawn from the market [1–3]. Animal studies are widely used for toxicity testing, but the predictions based on these assays are moderately successful [4]. Moreover, there is a growing awareness of the ethical objections related to animal testing, and efforts are being made to reduce the number of test animals required for toxicity testing [5,6]. Therefore, there is a high demand for new or improved methods predicting DILI well and early in the drug development process. This includes amongst others imaging technologies, omics technologies, advanced *in vitro* models and computational modelling [7,8].

Artificial intelligence and computational modelling approaches will likely be essential for improving toxicity predictions, reducing the need for test animals as well as for laborious *in vitro* experiments [5]. Moreover, mathematical modelling can help to gain a mechanistic understanding of biological processes [9]. Systems of ordinary differential equations (ODEs) are especially suitable for modelling temporal changes in complex biological systems, such as the modulation of adaptive cellular stress responses, because they can translate mechanistic processes into a mathematical framework and are easy and inexpensive to numerically simulate [10].

When cells are exposed to chemicals, such as drugs, this can cause cellular stress. This stress activates specific stress pathways [11] that mainly intend to regain homeostasis, but when the stress is too severe, it can lead to cell death (e.g. apoptosis or necrosis) and consequently lead to DILI when this occurs in the liver [12]. In the past years, mathematical models of various cellular stress pathways have been developed [13,14]. Often multiple of such pathways simultaneously become active upon drug exposure. This can be due to drugs activating more than one pathway yet also due to crosstalk between pathways. To fully understand why drug exposure in some cases leads to adverse reactions and to predict adversity, we thus need to combine pathways, including their interactions.

Two important pathways that can influence cell fate and consequently play a role in DILI include the DNA damage response (DDR) [15,16] and the oxidative stress response (OSR) [17,18]. The DDR activates when DNA damage occurs either spontaneously or due to exposure to chemicals, radiation or ultraviolet light [19]. Depending on the type and severity of the damage a distinct response takes place, which is mainly regulated by p53. p53 is phosphorylated by DNA damage sensing kinases or their downstream targets [20,21]. Following its phosphorylation, p53 acts as a transcription factor (TF) which leads to the transcription of many downstream targets such as p21, B-cell translocation gene 2 (BTG2) and mouse double minute 2 homolog (MDM2) [22]. MDM2 is a negative regulator of p53; complex formation of these proteins reduces the TF activity of p53 and promotes p53 degradation [23,24]. The interplay between MDM2 and p53 largely determines the dynamics of the DDR [24]. In the past decades, many different models have been developed to describe the DDR for varying applications and differing a lot in complexity [25,26]. Specifically, we previously developed an ODE model describing the cisplatin-induced DDR [27], based on HepG2 liver cells [27].

The second stress response pathway of interest, i.e., the OSR, reacts to oxidative stress which is caused by the accumulation of reactive metabolites (RM) or reactive oxygen species (ROS). OSR activation results in increased anti-oxidants, such as glutathione (GSH), to scavenge ROS or RM [28]. This protective pathway is activated by nuclear factor erythroid 2-like 2 (NRF2, gene name NFE2L2). NRF2 is constantly produced at a high rate and binds to Kelch-like ECH-associated protein 1 (KEAP1) in basal conditions [29–31]. Subsequently, NRF2 is ubiquitinated and degraded by the proteasome [31]. Upon oxidative stress, KEAP1 becomes modified and one of the two binding sites between KEAP1 and NRF2 dissociates as a latch, while the other remains bound as a hinge. [32–34]. Two-site binding is required for the rapid degradation of NRF2 [31,32]. Since oxidative stress causes one of the bounds to dissociate, this reduces the ubiquitination and thus NRF2 stabilises. Furthermore, NRF2 will translocate to and accumulate in the nucleus where it binds antioxidant response elements (AREs), which promote the transcription of various target genes such as sulfiredoxin 1 (SRXN1) and NAD(P)H quinone oxidoreductase 1 (NQO1) [35,36]. In the past years, several ODE models have been developed to describe chemically induced oxidative stress [37–39].

Previous studies have presented evidence of crosstalk between the OSR and DDR. Specifically, reported interactions include p21 inhibiting NRF2 degradation [40], phosphorylated p53 reducing the transcriptional activity of NRF2 [41], ROS causing DNA damage [42], NRF2-mediated transcription of MDM2 [43,44] and NQO1 stabilising p53 [45]. Computational modelling can help to indicate the key interactions among these and their importance in describing protein dynamics [46]. Moreover, when coupling stress pathway models to models predicting cellular adversity such as DILI, taking crosstalk into account could be essential for making proper predictions. With respect to OSR and DDR crosstalk, only one computational model has been created to study this interaction. Specifically, Pereira et al. [47] found that within the modelled p53-NRF2 signalling network the only crosstalk mechanism required to explain experimental measurements in breast-mammary epithelial cells included shared ROS induction. Because this model was only applied to stress induced by ROS, was developed for early time points only, and was developed for breast epithelial rather than liver cells, we need a different model for application to DILI predictions.

In the current study, we established mathematical crosstalk models between OSR and DDR. We developed a new model for the OSR pathway and employed a previously developed model for the DDR pathway by Heldring et al. [48]. The DDR model [48] was previously used to describe responses to DNA damaging agent cisplatin, and to extrapolate the responses between liver cell lines. We here applied the new OSR model and previously published DDR model to previously published liver HepG2 data of cellular responses to other compounds [49]. Next, we combined these two pathway models based on various potential modes of crosstalk found in the literature. Multiple variants of this p53-NRF2-pathway model differed in their abilities to describe chemically-induced stress pathway activation in HepG2 cells. Consequently, our models indicate the importance of the different modes of crosstalk and their effect on stress pathway activation.

## Results

### DDR model simulates activity in DNA damage reporters upon etoposide exposure

**Fig 1:**
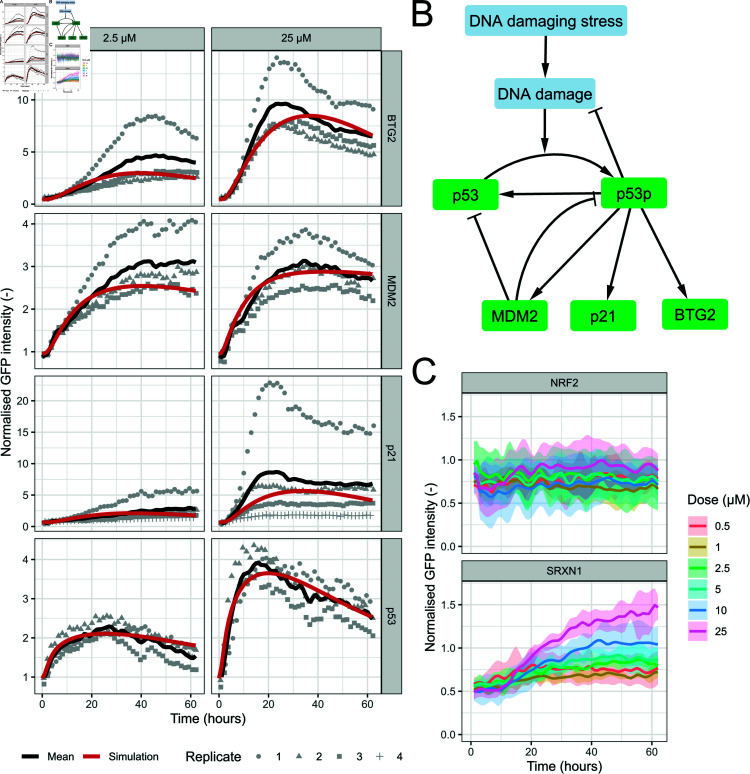
DDR model describes data from HepG2 cells exposed to etoposide. A) Simulations (red) of the DDR model for BTG2, MDM2, p21 and p53 combined with data (black line represents the mean, grey points the measurements per replicate) for these proteins after exposure to 2.5 and 25 µM etoposide. B) Schematic overview of the model for the DDR. C) Data for NRF2 and SRXN1 HepG2 reporter cell lines after exposure to six concentrations of etoposide. The colour represents the dose and the shaded area indicates the standard deviation across replicates.

We started by investigating previously published etoposide data from Wijaya et al. [49], who exposed HepG2 liver cells with integrated BAC-GFP reporters to this compound and monitored responses of the labelled DDR related proteins (p53, MDM2, p21, BTG2) over time. Etoposide is a topoisomerase II inhibitor, leading to double-strand DNA breaks [50]. As expected, the data of the HepG2 GFP-reporter cell lines exposed to etoposide exhibited a clear induction of the DDR (Fig 1A). Based on the measured cell viabilities (S1 Fig.), we selected six out of eight concentrations (i.e. 0.5, 1, 2.5, 5, 10 and 25 µM). We deemed the concentrations above 25 µM (i.e. 50 and 100 µM) as too toxic because the average cell count (S1A Fig) clearly decreased and the mean fraction of AnV-positive cells (S1B Fig) exceeded 0.15 during day 3 of the experiment. Note that the mean fraction of PI-positive cells remained below 0.05 (S1C Fig) and was not taken into account for the dose selection.

To simulate the DNA damage response, we used our previously published model [48] (Fig 1B; see Methods for details), which we originally developed to describe the DDR upon cisplatin exposure. The model parameters (see S1 Table.) were calibrated using data for the six selected concentrations of etoposide. The model described the data for p53, MDM2, BTG2 and p21 upon exposure to etoposide reasonably well (Figs 1A and S2), suggesting the general applicability of the model for DNA damaging compounds. The dose-response relationship is non-linear as can be seen from the model-estimated values for the initial stress levels for each concentration of etoposide (S3A Fig), which saturate with increasing etoposide dose.

When investigating the data of other available reporters (Fig 1C), we observed a dose-dependent upregulation in NRF2 target SRXN1 as well. A potential explanation is that there is crosstalk between DDR activation and OSR elements. In conclusion, the model described the etoposide-induced DDR well. The model does not contain any OSR components, even though we observe a clear induction of SRXN1 upon etoposide exposure. Therefore, adding OSR induction to this model could be a valuable extension.

### OSR model describes the early peak in NRF2 after exposure to DEM

Next, we investigated data of a typical oxidative stress inducer, diethyl maleate (DEM) [51,52]. Based on the cell viability data (S4 Fig.) we decided to include all eight doses available in the HepG2 data of Wijaya et al. [49] (i.e. 2, 10, 20, 40, 80, 120, 160, 200 µM). Indeed, even for the highest dose, the mean relative cell count did not decrease, the mean fraction of AnV-positive cells never exceeded 0.1, and the mean fraction of PI-positive cells remained below 0.03.

**Fig 2:**
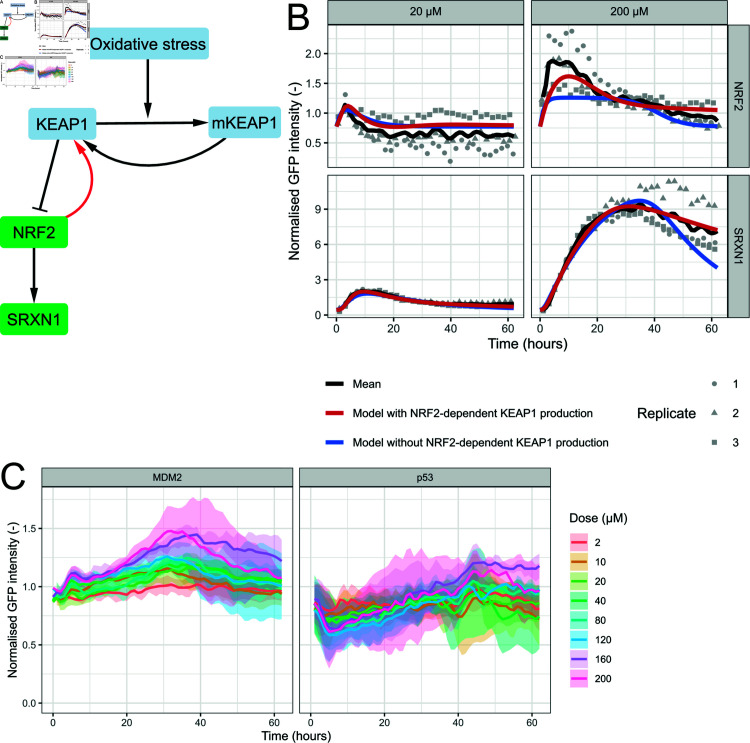
OSR model describes data from HepG2 cells exposed to DEM when NRF2 stimulates KEAP1 production. A) Schematic overview of the model for the OSR. B) Experimental data along with simulations of the OSR model with (red) and without (blue) NRF2-dependent KEAP1 production. The black line represents the mean, and the grey points the measurements per replicate after exposure to 20 and 200 µM DEM. C) Data for MDM2 and p53 after exposure to eight concentrations of DEM. The colour indicates the dose and the shaded area indicates the standard deviation.

We then developed a simplified model for the OSR (Fig 2A; see Methods for details) in order to properly simulate the NRF2 and SRXN1 data upon DEM exposure. We considered two model variants, one including NRF2-mediated stimulation of KEAP1 [53] and one without. Following model parameter calibration based on the 8 DEM concentrations (S2 Table.), the simulations of the model with NRF2-mediated KEAP1 regulation exhibited a similar dynamics as the data (Figs 2B and S5, red line). Note that the stimulating link from NRF2 to KEAP1 was required to describe the quick rise and decrease observed in NRF2 upon DEM exposure: A model without that link performed clearly worse, especially for high concentrations (Figs 2B and S5, blue line; model with NRF2-dependent KEAP1 production - AIC: -1836, BIC: -1714; model without NRF2-dependent KEAP1 production - AIC: -924, BIC: -818). As was the case for the DDR model following etoposide exposure, for the selected OSR model (i.e., with NRF2-mediated KEAP1 regulation) following DEM exposure the estimated values of the initial stress levels exhibited a non-linear dose-response relationship (S3B Fig).

The data of other HepG2 reporters exposed to DEM also exhibited interesting dynamics in both p53 and MDM2 (Fig 2C). Expression of p53 rapidly declined during the first 5–7 hours after exposure, followed by a gradual increase in expression. To check if the profound initial decrease was not caused by outlier replicates and/or concentrations, we investigated the the early time points of individual p53 replicates. However, all of the replicates show similar dynamics (S6 Fig.). Rather than exhibiting a decrease like p53, MDM2 initially increased for approximately 40 hours, succeeded by a gradual decrease. Downstream targets p21 and BTG2 also mildly responded to DEM, with an increase over time for both proteins and a later decrease for p21 (S7 Fig.).

Even though it is known that oxidative stress (due to e.g. reactive oxygen species (ROS)) can lead to DNA damage [42], this process cannot solely explain the experimentally observed dynamics. As shown in Fig 1, DNA damage generally leads to an initial increase in p53 followed by a decrease since MDM2 stimulates p53 degradation. Therefore, the initial decrease in p53 cannot be explained only by oxidative stress-induced DNA damage, suggesting other crosstalk plays a role as well. In conclusion, our model describes the OSR dynamics upon DEM exposure well, yet needs to be expanded with crosstalk to describe the behaviour of p53 and MDM2 as well.

### Activity of p21 is sufficient to explain OSR activation upon etoposide exposure

Our observation of an increase in SRXN1 expression upon exposure to etoposide in Fig 1C suggested crosstalk between DDR and OSR elements within HepG2 cells. Indeed, multiple interactions reported in literature could cause this increase in OSR activity, as proxied by SRXN1 in our case. Specifically, the presence of p21 can lead to an increase in NRF2 activity and its downstream targets [40]. This is caused by p21 competing with KEAP1 for binding to NRF2: When p21 binds to one of the two NRF2 binding sites, NRF2 stabilises because it cannot be ubiquitinated [40]. As a consequence, proteins downstream of NRF2, such as SRXN1 become highly expressed.

Another mode of DDR-OSR crosstalk that could be important is the reduction of transcriptional activity mediated by NRF2 via phosphorylated p53 [41]. Specifically, activated p53 can bind to promoter elements inhibiting transcription of NRF2 target genes. Since we observed upregulation of SRXN1, an inhibition of transcription of SRXN1 caused by p53 is unlikely. However, inhibition of KEAP1 transcription by phosphorylated p53 could potentially play a role because low expression of KEAP1 leads to little inhibition of NRF2, which in turn results in a high SRXN1 expression.

**Fig 3:**
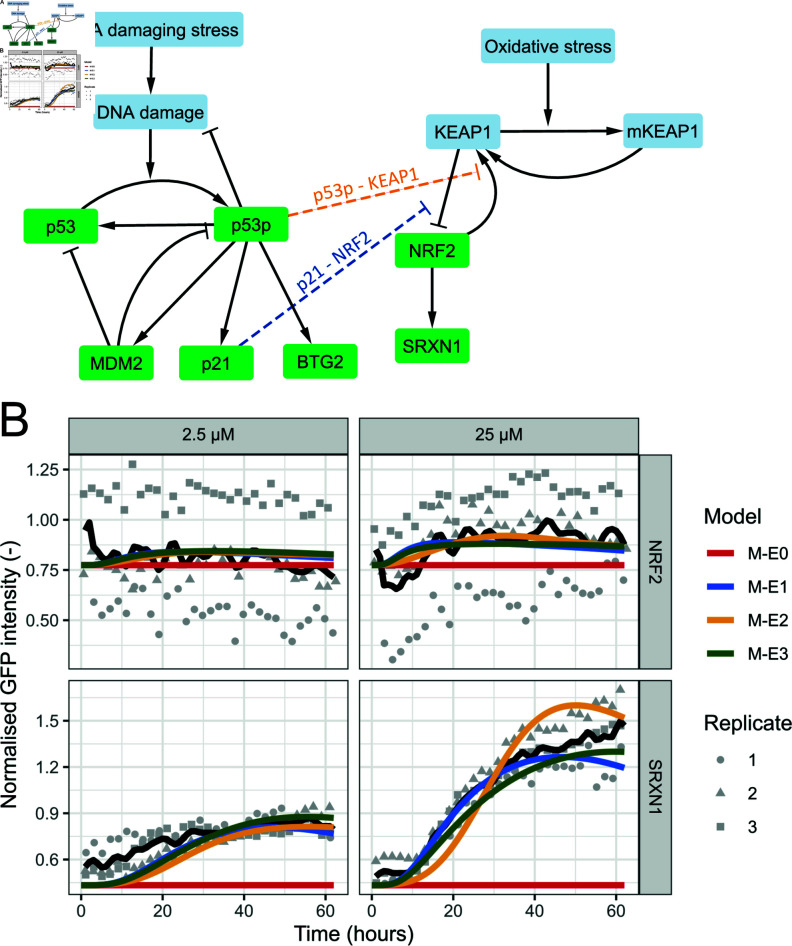
Crosstalk models to describe etoposide-induced OSR activity. A) Schematic overview of the model for the DDR and OSR upon DNA damage induction. Pathways are connected by crosstalk via p21 and p53p. B) Simulations of four different model versions (coloured lines) for NRF2 and SRXN1 combined with data (black line represents the mean, grey points the measurements per replicate) for these proteins after exposure to 2.5 and 25 µM etoposide.

We considered both modes of crosstalk separately, as well as jointly. Moreover, we considered etoposide itself not to induce oxidative stress. Model M-E1 contains the p21-mediated NRF2/SRXN1 expression (termed p21-NRF2), model M-E2 the p53-mediated KEAP1 inhibition (termed p53-KEAP1) and model M-E3 includes both modes of crosstalk (see coloured arrows in Fig 3A; equations in Methods). This resulted in a total of three variants of the DDR-OSR crosstalk model for etoposide. For each model variant, we only calibrated the extra crosstalk parameter(s) required for crosstalk (see new and modified values in S3 Table., the other values remain as in S1 Table. and column 4 of S2 Table.). Note that this also led to a change in the degradation parameter(s) of the modified equations, to ensure the model starts in steady state (see Methods for details).

Following parameter calibration to NRF2 and SRXN1 data upon etoposide exposure, all 3 DDR-OSR crosstalk model variants described the data reasonably well (Figs 3B and S8). Note that the decrease of the mean NRF2 data after the first timepoint is caused by the absence of data in the first 3 hours in replicate 1 that exhibits overall a slightly lower expression. Especially for low concentrations, simulations were highly similar across the 3 model variants. However, for high concentrations, differences were more apparent. Specifically, models M-E1 and M-E3 still resulted in similar fits, with model M-E3 remaining high at late time points which matched the experimental data well, while model M-E1 described the data at early time points well. Model M-E2 exhibited slightly different dynamics for SRXN1 than in the experimental data, when comparing the initial delay in SRXN1 increase, the subsequent steepness of the SRXN1 increase, and the SRXN1 behaviour during the last 10 hours. Based on these qualitative observations, selection of either model M-E1 or M-E3 was thus appropriate.

Apart from visually evaluating these models, we also compared the models quantitatively by computing the AIC and BIC. Model M-E1 had both the lowest AIC and BIC (Table 1) and could therefore be regarded as the best model. In conclusion, based on both qualitative observations (Figs 3 and S8) and statistical evaluation with AIC and BIC (Table 1), each of the three DDR-OSR crosstalk models is an improvement from the model without crosstalk. Model M-E1, including only p21-NRF2 crosstalk, was the most appropriate to describe this data based on information criteria. This analysis thus suggests that p21 forms the most prominent link by which DDR activity promotes OSR activity.

**Table 1:** Comparison of the quality of models describing etoposide exposure.

Model	Crosstalk	Equations	Fitted parameters	Cost (RSS)	AIC	BIC
M-E0	No crosstalk	2–8, 10, 12, 13	0	133.07	–2317.16	–2317.16
M-E1	p21-NRF2	2–8, 10, 13, 14	1	45.19	–3500.98	–3495.97
M-E2	p53p-KEAP1	2–8, 12, 13, 15	1	48.18	–3430.73	–3425.73
M-E3	p21-NRF2 & p53p-KEAP1	2–8, 13–15	2	45.62	–3488.56	–3478.55

The equations defining each model can be found in the Methods. The cost, AIC and BIC were determined based on the data for NRF2 and SRXN1 (1098 data points).

### NRF2-mediated MDM2 production combined with ROS-dependent DNA damage describes DDR activation upon DEM exposure

The observation that HepG2 cells exposed to DEM exhibited unexpected early responses in p53 and MDM2 dynamics (Fig 2C) suggested that besides ROS-induced DNA damage additional OSR-DDR crosstalk affected p53 and MDM2 dynamics. Therefore, we searched for other potentially relevant effects of oxidative stress proteins on the DNA damage pathway. One such interaction might be the NRF2-mediated transcription of MDM2, which has been reported in murine embryonic fibroblasts and pancreatic cells [43,44]. The quick increase in NRF2 upon DEM exposure could have led to a quick increase in MDM2 and consequently to increased degradation of p53.

Another interaction described in literature is the stabilization of p53 by NQO1 [45,54,55]. Like SRXN1, NQO1 is a downstream target of NRF2 and it binds and stabilizes p53 but does not block MDM2-dependent p53 degradation [56]. This interaction could help to explain the increase in p53 after approximately 20 hours. Since NQO1 is lacking in our OSR model we utilized SRXN1 as a surrogate for NQO1. A gene coregulation network-based analysis of transcriptomics data in HepG2 clustered SRXN1 and NQO1 in the same network module [57]. This indicates that SRXN1 and NQO1 are in general coregulated, and likely exhibit similar dynamics.

**Fig 4:**
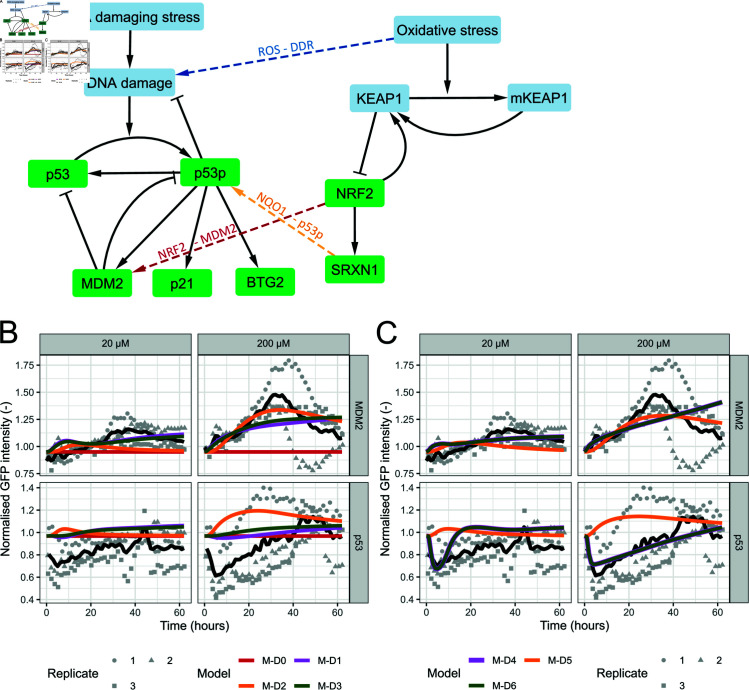
Crosstalk models to describe DEM-induced DDR activity. A) Schematic overview of the model for the DDR and OSR upon oxidative stress. Pathways are connected by crosstalk via oxidative stress, NRF2 and SRXN1. B) & C) Different model versions without (B) and with (C) adapted MDM2-dependent p53 degradation. Simulations (coloured lines) for MDM2 and p53 combined with data (black line represents the mean, grey points the measurements per replicate) for these proteins after exposure to 20 and 200 µM DEM.

We included several combinations of the three mentioned interactions (termed ROS-DDR for ROS-induced DNA damage, NRF2-MDM2 for NRF2-mediated MDM2 production, and SRXN1-p53 for SRXN1 (NQO1)-mediated p53 stabilization) in models M-D1, M-D2 and M-D3 (S4 Table.; for equations see Methods). All model variants visually improved the description of the experimental data compared to absence of OSR-DDR crosstalk (Figs 4B and S9A), which was supported by AIC and BIC statistics (Table 2). Nevertheless, the result was not yet satisfactory, primarily because none of the simulations led to an early p53 decline (Fig 4B).

**Table 2:** Comparison of the quality of models describing DEM exposure.

Model	Crosstalk	Equations	Fitted parameters	Cost (RSS)	AIC	BIC
M-D0	No crosstalk	3–13	0	100.46	–6014.24	–6014.24
M-D1	NRF2-MDM2 & ROS-DDR	4, 5, 7–13, 16, 17	5	77.29	–6530.73	–6502.71
M-D2	NRF2-MDM2 & SRXN1-p53	3, 5, 7–13, 17, 18	4	92.56	–6170.70	–6148.28
M-D3	NRF2-MDM2, ROS-DDR & SRXN1-p53	5, 7–13, 16–18	6	78.92	–6486.93	6453.30
M-D4	Adapted MDM2-p53 degradation, NRF2-MDM2 & ROS-DDR	7–13, 16, 17, 19, 20	11	63.45	–6914.82	–6853.17
M-D5	Adapted MDM2-p53 degradation, NRF2-MDM2 & SRXN1-p53	3, 7–13, 17, 20, 21	10	88.65	–6245.30	–6189.25
M-D6	Adapted MDM2-p53 degradation, NRF2-MDM2, ROS-DDR & SRXN1-p53	7–13, 16, 17, 20, 21	12	63.47	–6912.31	–6845.05

The equations defining each model can be found in the Methods. The cost, AIC and BIC were determined based on data for p53 and MDM2 (2008 data points).

In order to understand why a strong initial p53 decline was not observed for our model variants, we studied the impact of MDM2-related parameters on p53. This analysis showed that for the current parameter values, only an extremely high level of MDM2 would lead to the desired p53 decline. For instance, when we simulated model M-D3 with an 8-fold increase in the maximal speed of NRF2-dependent MDM2 production, the correspondence to p53 data improved but this came at the expense of the MDM2 description (S10 Fig.). Since the increase in MDM2 is only modest in the experimental data, we hypothesised that the MDM2-dependent p53 interaction might be different in the setting with DEM-induced DDR compared to the etoposide-induced DDR. Therefore, we replaced the p53-MDM2 interaction term in the models with a Hill equation such that MDM2-induced p53 degradation achieves a maximum rate at high MDM2 concentrations, leading to models M-D4, M-D5, and M-D6. After recalibrating the corresponding parameters simultaneously with calibrating different combinations of crosstalk (see S5 Table.), models M-D4 and M-D6 managed to describe the initial decrease and late increase, while model M-D5 did not (Figs 4C and S9B; lines for M-D4 and M-D6 almost overlap). Consistently, the cost values and associated AIC and BIC values were lowest for these model variants (Table 2). Since Model M-D4 has fewer parameters and lower AIC/BIC than model M-D6, we took this as the preferred model. In conclusion, following inclusion of Hill-type p53-MDM2 interactions, the presence of NRF2-mediated MDM2 production and ROS-mediated DNA damage are sufficient to explain the observed DDR.

### *In silico* knockdowns predict strong effect of p21 on OSR activity

Following selection of models M-E1 and M-D4 as best models for etoposide and DEM exposure respectively, we noted that the selected models do not properly represent each of the individual replicates due to inter-replicate variability. We therefore investigated whether slight variation of model parameters could explain this variability by performing a sensitivity analysis (see Methods). Indeed, the effect of some parameters on the simulations of M-E1 (S11A Fig) and M-D4 (S12A Fig) was large. Varying the parameters with the most profound effect on the simulations by 20% helped to describe most of the variability between replicates (S11B and S12B Figs). Describing the variability was most successful for the directly induced proteins (i.e., p53 and MDM2 for etoposide, and NRF2 and SRXN1 for DEM).

Following the development of two separate models describing either DEM exposure causing DDR activity or etoposide exposure causing OSR activity, we next wanted to combine the different crosstalk mechanisms into one model. In principle, the slight OSR-mediated activation of the DDR upon DEM exposure might also feedback onto the OSR, and vice versa for etoposide exposure. Nevertheless, this secondary feedback might be too low to be biologically relevant in practice.

Ideally, we would have combined both models into one single model. However, the presence of compound-specific model elements such as the adapted term for MDM2-dependent p53 degradation precluded this. Therefore, we developed model M-CD for the combined crosstalk model upon DEM exposure and M-CE for the combined model upon etoposide exposure (without refitting or changing parameters). We hypothesised that the results would be very similar to models M-D4 and M-E1 respectively because the indirect activation of a pathway would be too minor to in turn affect the dynamics of the initially activated pathway.

**Fig 5:**
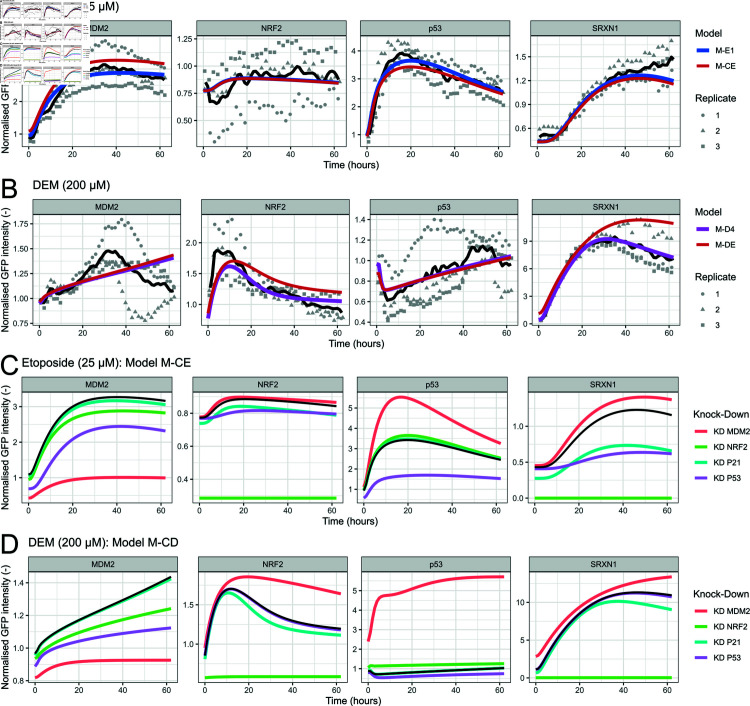
*In silico* knockdown predictions on the basis of crosstalk models. A-B) Simulation of best crosstalk model versions (blue or pink) and combined crosstalk models (red) for exposure to 25 µM etoposide (A) or 200 µM DEM (B). Simulations (coloured lines) and data (black line represents the mean, grey points the measurements per replicate) are shown for MDM2, NRF2, p53 and SRXN1. C-D) Simulations with (coloured lines) or without knockdowns (black line) for MDM2, NRF2, p53 and SRXN1, for exposure to 25 µM etoposide (C) or 200 µM DEM (D).

Figs 5A, 5B and S13 show the results for models M-CE and M-CD. As expected, both for the comparison of models M-CE and M-E1 upon etoposide exposure (Figs 5A and S13A) and of models M-CD and M-D4 upon DEM exposure (Figs 5B and S13B) the results are indeed similar. The largest deviation occurs in the simulation of SRXN1 upon DEM exposure, yet the description of the data is still reasonable.

In order to further quantify the importance of the crosstalk mechanisms between OSR and DDR, we used the models M-CE and M-CD to perform *in silico* knockdowns. Specifically, we determined the expected effect of knocking down NRF2, p53, MDM2 and p21 in combination with exposure to either etoposide or DEM. To simulate knockdowns we multiplied the modelled production terms (both basal production and production dependent on other variables) by 0.3, i.e. a 70 % reduction (see Methods for details).

The knockdown predictions for the two models show similar trends (Figs 5C, 5D, S14 and S15): An MDM2 knockdown leads to a large increase in p53 and also to an increase in NRF2 and SRXN1, caused by the increase of p21 downstream of p53. In the results for model M-CD the increases are more profound than in those for model M-CE. A knockdown of NRF2 leads to a strong decrease in SRXN1, a smaller but clear decrease in MDM2 and consequently a slight increase in p53 in both models. A knockdown in p21 hardly affects MDM2 and p53 but does lead to a substantial decrease in NRF2 and SRXN1 (especially in M-CE). Lastly, the p53 knockdown has a similar but less strong effect (compared to p21 knockdown) on NRF2 and SRXN1. As expected, a p53 knockdown also leads to a decrease in MDM2. In conclusion, our models predict that knockdowns within either OSR or DDR have a limited to moderate effect on protein expression in the connected pathway via crosstalk. Especially p21 is expected to substantially affect OSR activity.

## Discussion

Cells exposed to toxicants, such as drugs, activate a wide variety of stress pathways, often simultaneously. While computational models for individual pathways exist, we aimed to develop models to describe the dynamics of both OSR and DDR proteins simultaneously in HepG2 cells upon exposure to either etoposide or DEM. These models gave insight into the crosstalk between the two stress pathways. Our approach involved developing a simple model for the OSR pathway and adapting a previously developed model for the DDR pathway [48], and applying them to previously published data of Wijaya et al. [49]. Importantly, our models mainly contain key variables for which we had experimental data available, which implies that the models are simplified and do not include some of the relevant DDR and OSR processes. However, this approach allowed for parameter identification and provided us with comprehensible and computationally cheap models. Moreover, these base models successfully described the dynamics of key pathway proteins upon exposure to either DEM or etoposide. In the future, our models can be supplemented with additional processes taking place during the DDR and OSR, rendering a realistic description of the true complexity of these pathways.

Next, we coupled the two base pathway models, considering various potential modes of crosstalk found in the literature, considering the two crosstalk directions individually. By selecting the most suitable models to describe the data based on information criteria we managed to pinpoint which crosstalk mechanisms were relevant for HepG2 cells. For both etoposide and DEM exposure, this resulted in models decently describing the primary pathway activation as well as the secondary responses caused by crosstalk.

The sensitivity analysis that followed showed that varying certain parameters by 20% can largely explain the variability between replicates. This approach was most successful in describing the variability in the directly induced proteins (i.e., p53 and MDM2 upon etoposide exposure, and NRF2 and SRXN1 upon DEM exposure). For indirectly induced proteins, this approach was less successful: the absence of direct stress likely causes the parameters to have only a small effect. Rather, experimental noise is relatively large for these proteins as they have a lower intensity than upon direct induction.

To further explore the impact of the crosstalk, we included crosstalk in both directions in each of the models and performed *in silico* knockdowns. Both models led to similar trends upon knockdown, i.e., the same proteins increased or decreased compared to the situation without knockdown, providing confidence in our model-based expectations. However, the relative change for each protein differed between the simulation for DEM or etoposide exposure. The most profound effect of the crosstalk occurred during simulations of p21 knockdown, which led to a clear decrease in SRXN1 and NRF2.

To investigate the congruence between our models’ knockdown predictions and experimental data, we compared our predictions to results of Hiemstra et al. [58]. Upon knockdown of p53, Hiemstra et al. [58] observed only a slight difference in SRXN1 after exposure to 10 or 100 µM DEM. Similarly, in our simulations a knockdown of p53 hardly affected SRXN1 for either concentration (S14B Fig). Furthermore, when Hiemstra et al. [58] measured p21 expression after knockdowns combined with exposure to etoposide, NRF2 knockdown hardly changed p21 but as expected p53 knockdown did decrease the p21 intensity significantly. This is in accordance with our simulations of p21 after etoposide exposure and NRF2 or p53 knockdown (S15 Fig.): The p53 knockdown had a strong decreasing effect, while the NRF2 knockdown had a slight increasing effect. Still, minor quantitative differences between earlier published data and our model predictions occur, partly because our models are simplifications, but also because our rough estimate of the knockdown efficacy ( ∼  70 % reduction of production rates) is uncertain. Nevertheless, the overall similarities between the *in silico* and *in vitro* knockdown results, indicate that we captured the main biological connections with our models. Future knockdown data, acquired in various reporter cell lines would be of value to test all our *in silico* predictions and to evaluate model validity.

During the model selection process, we selected models that excluded some crosstalk interactions found in the literature, because these mechanisms did not sufficiently improve the description of the HepG2 data. Specifically, we omitted the p53p-mediated inhibition of NRF2 transcriptional activity from the etoposide model, and the inhibition of p53 degradation via NQO1, from the DEM model. This might suggest that these modes of crosstalk are not that relevant for describing the HepG2 protein dynamics. However, we want to emphasise that the conclusions about the existence and relevance of crosstalk interactions heavily depend on the dataset that we used, which had limitations. For instance, for the first omitted interaction, i.e., the reduction in transcriptional activity mediated by NRF2 caused by p53p, the AIC/BIC analysis indeed led to our model selection decision not to include the interaction in our final model for etoposide. However, the dynamics of model M-E3 qualitatively matched the data slightly better than model M-E1 in terms of absence of SRXN1 decrease at late time points (Figs 3 and S8). If data at time points well beyond 60 hours had been available, the AIC/BIC analysis might have led to a quantitative preference for model M-E3 over M-E1.

The redundancy of the p53p-mediated crosstalk could be due to only limited transcriptional activity mediated by NRF2 in our data, compared to the results of Faraonio et al. [41]. Even though HepG2 cells upregulated NRF2 only slightly, a clear response in SRXN1 occurred. If the transcription of ARE genes were reduced, we would expect the transcription of SRXN1 to be affected as well. Faraonio [41] found the largest decrease in transcriptional activity when cells were exposed to both DEM and cisplatin, directly leading to oxidative stress and DNA damage, respectively. This could explain why the crosstalk effect is less profound when there is only DNA damage stress. Consequently, it might be required to reintroduce p53p-mediated inhibition of NRF2 activity in the models when applying them to a situation with both types of stress. Furthermore, Chen et al. [59] reported that p53 can have both a stimulating and an inhibiting effect on NRF2, depending on the amount of p53. Thus, the p53p-NRF2 interaction may be more complicated than only stimulation, which would require a different implementation in our models to correctly describe diverse biological settings.

Besides p53p-mediated NRF2 activity, other crosstalk mechanisms could play a role in the activation of the OSR as well. One of these mechanisms could be the activation of NRF2 via BTG2. Specifically, BTG2 mediates upregulation of antioxidant enzymes known to be regulated by NRF2 in MCF7 cells [60]. BTG2 may act as a transcriptional co-activator for NFE2L2 but the effect could also be NRF2-independent. Even though model M-E1 is already able to describe the data well, these prior findings warrant further investigation of these interactions in HepG2 cells. Furthermore, etoposide was recently reported to induce oxidative stress in bacteria (Pseudomonas aeruginosa) [61] and DNA damage has been reported to induce ROS in both yeast and mammalian cells [62,63]. Even though it is unclear if this also happens in HepG2 cells, it challenges our assumption that etoposide exposure does not lead to oxidative stress. Furthermore, other signalling pathways could be involved in the crosstalk between DDR and OSR. For example, NF-*κ*b is known to have both pro- and anti-oxidative targets [64,65] and to be activated in the DNA damage response [66,67]. Similarly, JNK activation and redox signalling is linked to DNA damage [67,68] and consequently affects OSR activation [69,70]. Additional experiments should further elucidate the mentioned processes and could confirm if it is required to expand our model with etoposide-driven ROS production or additional signalling pathways.

With respect to crosstalk from OSR to DDR, model fits did not improve upon inclusion of p53 stabilization via NQO1. Here, one potential caveat is the replacement of NQO1 by SRXN1 in our model implementation, which was required due to the lack of NQO1 data. Since NQO1 and SRXN1 may exhibit differences in their dynamics, it is possible that including the NQO1 crosstalk in the model via SRXN1 prohibited improvement of fit quality. It should also be noted that the basal level of NQO1 in HepG2 and other hepatocellular carcinoma cells is relatively high [71,72]. Consequently, the effect of NQO1 might not be captured correctly by taking SRXN1 as an NQO1 proxy. The impact of NQO1 on p53 could thus be more important in other, non-cancerous, cell lines. Additional data on the protein dynamics of NQO1 would help to evaluate the importance amongst OSR-DDR crosstalk.

Based on information criteria analysis, we selected model M-D4 as appropriate model to describe the induction of p53 and MDM2 upon DEM exposure (Fig 4 and Table 2). However, the description of these DDR data could be further improved: Unlike the data, the simulations of MDM2 for high DEM concentrations, are increasing rather than decreasing after approximately 30 hours. Initially, we expected the decrease of MDM2 would be due to the rapid decrease in NRF2, since NRF2 is one of the stimulators of MDM2 in this model. However, this did not happen in the model, either because the increase in p53p compensated for the decrease in NRF2 or because the effect of NRF2 on MDM2 is only small. Refitting the parameters for the MDM2 production might lead to a closer match, but this would also worsen the simulations for exposure to etoposide.

Besides a crosstalk effect of NRF2 on MDM2, alternative mechanisms may explain high MDM2 degradation at late time points, as observed experimentally. DNA damage can lead to increased auto-degradation of MDM2 [73]. Since oxidative stress can induce DNA damage [42], as is included in our model, MDM2 autodegradation could be a reason for the decrease in MDM2 levels at late time points. One might expect this effect to also apply to the data for etoposide, where MDM2 only increased. However, the levels of p53 are very high following exposure to etoposide, and the associated high production rate of MDM2 might compensate for an increased auto-degradation. Therefore, we cannot exclude that MDM2 auto-degradation plays a role during the DEM-induced DDR.

A complicating factor in improving the p53/MDM2 part of the model is the negative feedback loop between MDM2 and p53. Changing MDM2 affects p53 and vice versa, which makes it difficult to adapt the model for both proteins simultaneously. Indeed, the relation between MDM2 and p53 is probably more complex than we currently included in the model, as evident from the difficulty to properly simulate the initial decrease in p53 upon exposure to DEM. We achieved this by adding compound-specific MDM2-dependent p53 degradation, which suggests that additional processes play a role in this difference in interaction between p53 and MDM2 across compounds. One complicating factor in quantifying the effect of MDM2 on p53 is the existence of MDMX (also known as MDM4), a homolog of MDM2 [74]. MDMX modulates the MDM2-dependent degradation of p53, and MDM2 and MDMX mutually depend on each other for the regulation of p53 [75]. Moreover, the regulation of both MDM2 and MDMX is complex: Even though both are transcriptionally activated by p53, they are also differentially regulated in many ways [76]. Thus, additional data on MDMX are required to understand if and how much effect it has in the settings studied here.

In general, model simplification by adapting the value of parameters depending on the compound, is a straightforward and potentially useful solution to obtain proper simulations of biological data. In this case, changing a few parameter values helped to describe the initial decrease in p53 correctly upon DEM exposure. However, such an approach also comes with disadvantages: Due to the compound-specific parameters in our models, we could not combine the two separate models into a single joint model. Moreover, having compound-specific parameters implies that such parameters need to be recalibrated when applied to data for new compounds unless the compound has highly similar effects as a compound previously used for parameter calibration. This reduces the applicability and generalisability of models. Therefore, further research into the mechanisms leading to compound specificity in parameters is highly desirable.

Our models are applicable in a certain domain: for specific concentrations of DEM and etoposide, they can describe the response for up to 60 hours. In future research, it would be relevant to investigate how well these models perform outside these conditions. By generating a dataset for different concentrations or longer timescales, one could investigate the extrapolation capacity of these models. It should be noted that the 2D cell culture conditions for these experiments preclude imaging far beyond 60 hours. Hence, 3D spheroid set-ups are a more appropriate setting for testing of our models’ applicability domain [77,78]. Moreover, investigating whether different OSR or DDR compounds could be described by these models would be an interesting expansion. Based on previous research where models were applied to different OSR-invoking compounds [39] and our experience in translating a model developed for cisplatin [27] to etoposide, we do not expect major changes in the model structure (in other words, in molecular interactions) but at least some changes in the parameter values are likely required (i.e., the magnitude of the interactions).

We do not expect major problems when expanding these models to different concentrations in the adaptive region, as we already applied them to 6 or 8 different doses of etoposide or DEM. Using the curves for the estimated initial stress levels (S3 Fig.), one could extrapolate the required stress value for the desired dose. However, for much higher concentrations we do not expect our models to be valid anymore. We specifically developed our models to describe the adaptive response to stress, when cells can still recover. When many cells die the average stress pathway activation in that population is expected to go down because a large cellular fraction will be dying and become unresponsive at stress pathway level [79], which affects the response observed in the population. This problem might be circumvented by investigating responses on a single-cell level, which would require a modified experimental design to enable single-cell tracking over time. In such efforts, we recommend combining investigation of stress pathways with death pathways that become substantially activated for high compound doses. This would provide quantitative insight in this interplay and a comprehensive view on DILI.

Another unresolved question is how well our models would perform to describe the dynamics in different cell types. We developed the models based on data in HepG2 cells. HepG2 cells are a commonly used cell type to represent liver cells but it is a carcinoma cell line and therefore not fully representative for hepatocytes. Among other things, they proliferate continuously and have low levels of metabolising enzymes [80]. Hepatocyte-like cells (HLC) differentiated from human-induced pluripotent stem cells are considered more similar to human liver tissue than HepG2 cells, although not as similar as Primary Human Hepatocytes (PHH) [81]. Unfortunately, time-dynamic protein data is not yet available for these cell types, although recently developed HLC reporter cell lines allow for such dynamic data [82]. Such data can be used in the future to apply our models to. We expect our models to be applicable to different liver cell types since etoposide activates the DNA damage response also in PHH [83] and DEM induces the oxidative stress response also in PHH [83,84] and HLC [82].

As mentioned before, cellular responses to stress are much more complex than considered within the models we have developed during this study. Despite several limitations, our models can simulate most of the data on the activation of both OSR and DDR-related proteins caused by exposure to DEM or etoposide. Moreover, the modelling implicated plausible key players in the regulation of crosstalk between the OSR and DDR pathways. Furthermore, *in silico* knockdown predictions largely agreed with previously published experimental data, indicating model validity. Future research integrating experimental data into computational models will be required to further elucidate the (crosstalk between) OSR and DDR pathways. Nevertheless, our current models could already be connected to other models that predict cell fate or adversity based on expression of specific proteins. In this way, our models can play a valuable role in the prediction of adverse reactions such as DILI.

## Methods

In order to develop our p53-NRF2 pathway models, we used previously published data of Wijaya et al. [49]. In brief, Wijaya et al. [49] performed a screen with HepG2 BAC-GFP reporter cell lines [85] exposed to eight concentrations of various compounds. They used live fluorescence-based imaging of reporter activity for 60 hours with a time interval of 90 (BTG2, MDM2, p21 and p53) or 120 (NRF2 and SRXN1) minutes to monitor abundance of relevant proteins in the DDR and OSR pathways. Here, we use the data generated upon exposure to topoisomerase II inhibitor etoposide and oxidative stressor diethyl maleate (DEM) with the BTG2, MDM2, NRF2, p21, p53 and SRXN1 cell lines. For a detailed description of the experimental procedures, we refer to the Methods section in [49].

### Data analysis

#### Viability data

To select suitable concentrations for our modelling, we used the cell viability data generated by Wijaya et al. [49]. Specifically, we analysed the cell count, Annexin V (AnV) and Propidium Iodide (PI) measurements of all 11 reporters used in the screen (i.e. ATF4, BIP, CHOP, XBP1, NRF2, SRXN1, HMOX1, p53, MDM2, BTG2 and p21) that were exposed to DEM and etoposide. For each reporter, at least 3 replicates were available (for p21 4 replicates), resulting in a total of 34 time-course measurements that were used to compute the cell count, PI and AnV data for each treatment. To account for differences in the number of cells at the start of the experiment, we divided the number of cells by the number of cells at the first time point, i.e., each well started with a relative cell count of 1. To evaluate the amount of cell death, we used the fraction AnV- or PI-positive cells. A cell is regarded as AnV- or PI-positive when the AnV or PI area is at least 10 per cent of the total 2-pixel dilated nucleus area. Based on a decreasing cell count and a fraction of AnV-positive cells exceeding 0.15 for the two highest concentrations of etoposide, we omitted this subset of the data for model calibration.

#### GFP data

Using the green fluorescent protein (GFP) intensities obtained after image analysis by Wijaya et al. [49] as representative for the abundance of labelled proteins, we calculated the geometric mean of the GFP measurements of all individual cells per image. Similarly, we also obtained the GFP intensity for wells treated with 0.01 µM DMSO in this way. This control value corresponding to the same plate and time point was subtracted from the GFP intensity of each treatment to correct for background signal.

Next, we applied further normalisation to the background-corrected data using the following formula:


$$\begin{array}{c} x_{normalised}=\frac{x-x_{min}}{x_{mean}-x_{min}}\;, \end{array}$$ (1)

with *x* a measured value, $x_{min}$ the minimally measured value among all treatments (see Table 1 in [49] for an overview) and time points of that plate, and $x_{mean}$ the average value for the GFP intensity of all treatments and time points for that plate. With this approach, we obtained a relatively uniform scale between the replicates, avoiding potential major influence of one outlier that shifts all other values, which can happen for the commonly used min-max normalisation.

For every plate, the measurements were performed at slightly different time points due to the time required for experimental procedures. Interpolation of the data ensured suitability for our parameter fitting process (explained below). We interpolated the data to time points in the interval 0 to 62 hours with time steps of 1 hour with a natural cubic spline using the spline function of the stats package in R [86]. To obtain values for the initial time point(s), the data had to be extrapolated, because the initial measurements did not occur exactly at time point 0. We used the same natural spline approach for extrapolation but only used extrapolated values within 0.5 hours of the first measured time point to make sure the generated estimates would be close to the initial measurement. The mean of the extrapolated GFP intensities at the initial time point for each state variable was used as the initial state value for the associated model variable (see model explanation below) in the simulations. As for the AnV, PI, and cell count data, for p21 there were 4 replicates available and for all other reporters, the data contained 3 replicates.

### Mathematical modelling

#### Base model DDR

We used the DDR model of Heldring et al. [48] as base for the combined p53-NRF2 model designed in the current study. Compared to that model, we discarded the scaling and offset parameters, changed the initial states corresponding to the values in the etoposide data and recalibrated the parameters. The model contains seven state variables: $S_{DD}$ (DNA damage inflicting stress), *DD* (DNA damage), $p_{53}$ (unphosphorylated p53 protein), $p_{53p}$ (phosphorylated p53 protein), $M_{2}$ (MDM2 protein), $B_{2}$ (BTG2 protein) and $p_{21}$ (p21 protein)). The total p53, which is what the p53 reporter measures, is the sum of $p_{53}$ and $p_{53p}$. The base model consists of the following system of equations:


$$\begin{array}{c} \frac{dS_{DD}}{dt}=-\tau_{D}\cdot S_{DD}\;, \end{array}$$ (2)

$$\begin{array}{c} \frac{dDD}{dt}=b_{DD}+S_{DD}-d_{DD,p_{53p}}\cdot p_{53p}\cdot DD\;, \end{array}$$ (3)

$$\begin{array}{c} \frac{dp_{53}}{dt}=b_{p_{53}}+r_{dp}\cdot p_{53p}-r_{p}\cdot p_{53}\cdot DD-d_{p_{53}}\cdot \left(1+d_{p_{53},M_{2}}\cdot M_{2}\right)\cdot p_{53}\;, \end{array}$$ (4)

$$\begin{array}{c} \frac{dp_{53p}}{dt}=r_{p}\cdot p_{53}\cdot DD-r_{dp}\cdot p_{53p}-d_{p_{53p}}\cdot \left(1+d_{p_{53p},M_{2}}\cdot M_{2}\right)\cdot p_{53p}\;, \end{array}$$ (5)

$$\begin{array}{c} \frac{dM_{2}}{dt}=b_{M_{2}}+\frac{V_{M_{2}}\cdot p_{53p}^{4}}{Km_{M_{2}}^{4}+p_{53p}^{4}}-d_{M2}\cdot M_{2}\;, \end{array}$$ (6)

$$\begin{array}{c} \frac{dB_{2}}{dt}=b_{B_{2}}+\frac{V_{B_{2}}\cdot p_{53p}^{4}}{Km_{B_{2}}^{4}+p_{53p}^{4}}-d_{B_{2}}\cdot B_{2}\;, \end{array}$$ (7)

$$\begin{array}{c} \frac{dp_{21}}{dt}=b_{p_{21}}+\frac{V_{p_{21}}\cdot p_{53p}^{4}}{Km_{p_{21}}^{4}+p_{53p}^{4}}-d_{p_{21}}\cdot p_{21}\;. \end{array}$$ (8)

In this system, $\tau_{D}$ is the decay rate for the DNA damage stress, $b_{X}$ is the basal production rate for state variable *X*, $d_{X}$ is the basal degradation rate of state variable *X*, $d_{X,M_{2}}$ is the factor by which MDM2 promotes degradation of *X*, $r_{p}$ is the phosphorylation rate of p53, $r_{dp}$ is the dephosphorylation rate of p53p, $V_{X}$ is the maximal speed of *X* production (which depends on TF p53p), and $Km_{X}$ is the amount of TF (i.e., p53p in this model) for which the production rate of *X* is half-maximal. Note that the Hill exponent for the non-linear production of p53 targets is set to 4, because phosphorylated p53 organizes in tetramers before binding to DNA.

#### Base model OSR

We developed a simplified model of NRF2 signalling as a base for the subsequent combined p53-NRF2 model. The base NRF2 model contains the five state variables $S_{OS}$ (oxidative stress), $K_{1}$ (KEAP1 protein), $K_{1mod}$ (modified KEAP1 protein), $N_{2}$ (NRF2 protein) and $S_{1}$ (SRXN1 protein), and consists of the following equations:


$$\begin{array}{c} \frac{dS_{OX}}{dt}=-\tau_{O}\cdot S_{OX}\;, \end{array}$$ (9)

$$\begin{array}{c} \frac{dK_{1}}{dt}=b_{K_{1}}+\frac{V_{K_{1}}\cdot N_{2}^{n_{K_{1}}}}{Km_{K_{1}}^{n_{K_{1}}}+N_{2}^{n_{K_{1}}}}-r_{m}\cdot S_{OX}\cdot K_{1}+r_{um}\cdot K_{1mod}-d_{K_{1}}\cdot K_{1}\;, \end{array}$$ (10)

$$\begin{array}{c} \frac{dK_{1mod}}{dt}=r_{m}\cdot S_{OX}\cdot K_{1}-r_{um}\cdot K_{1mod}-d_{K_{1}}\cdot K_{1mod}\;, \end{array}$$ (11)

$$\begin{array}{c} \frac{dN_{2}}{dt}=b_{N_{2}}-\frac{Vd_{N_{2}}\cdot K_{1}\cdot N_{2}}{Km_{N_{2}}+N_{2}}-d_{N_{2}}\cdot N_{2}\;, \end{array}$$ (12)

$$\begin{array}{c} \frac{dS_{1}}{dt}=b_{S_{1}}+\frac{V_{S_{1}}\cdot N_{2}^{n_{S_{1}}}}{Km_{S_{1}}^{n_{S_{1}}}+N_{2}^{n_{S_{1}}}}-d_{S_{1}}\cdot S_{1}\;. \end{array}$$ (13)

In these equations, as in the DDR model, $b_{X}$ is the basal production for state variable *X* and $d_{X}$ is the basal degradation rate of state variable *X*, $V_{X}$ is the maximal speed of *X* production (which depends on NRF2 here), and $Km_{X}$ is the amount of TF (i.e., NRF2 in this model) for which the production or degradation rate of *X* is half-maximal. Furthermore, $\tau_{O}$ is the decay rate for the oxidative stress, $r_{m}$ is the (stress-dependent) modification rate of KEAP1, $r_{um}$ is the unmodification rate of modified KEAP1, $Vd_{N_{2}}$ is the maximal KEAP1-dependent degradation rate of NRF2, $n_{X}$ is the Hill coefficient for the non-linear production of *X*.

#### Crosstalk model etoposide

To develop our crosstalk models, we combined the models of individual pathways described above and included several modes of crosstalk. With our first set of crosstalk models, aiming to describe the cellular response to etoposide, we focused on the impact of the DDR on the OSR (but not vice versa). Therefore, we included two modes of crosstalk suggested by literature. The first one describes p21-mediated inhibition of the KEAP1-dependent degradation of NRF2 [40], which we implemented by replacing with


$$\begin{array}{c} \frac{dN_{2}}{dt}=b_{N_{2}}-\frac{Vd_{N_{2}}\cdot K_{1}\cdot N_{2}}{(Km_{N_{2}}+C_{p_{21}}\cdot p_{21})+N_{2}}-d_{N_{2}}\cdot N_{2}\;, \end{array}$$ (14)

with $C_{p_{21}}$ as a new parameter representing the strength of p21-mediated inhibition. The second mode of crosstalk is the inhibition of NRF2-dependent production of KEAP1 via p53p [41], which we implemented by replacing with


$$\begin{array}{c} \frac{dK_{1}}{dt}=b_{K_{1}}+\frac{V_{K_{1}}\cdot N_{2}^{n_{K_{1}}}}{{\left(Km_{K_{1}}+C_{p_{53p}}\cdot p_{53p}\right)}^{n_{K_{1}}}+N_{2}^{n_{K_{1}}}}-r_{m}\cdot S_{OX}\cdot K_{1}+r_{um}\cdot K_{1mod}-d_{K_{1}}\cdot K_{1}\;, \end{array}$$ (15)

with new parameter $C_{p_{53p}}$, representing the strength of p53p-mediated inhibition.

Moreover, we removed the variables for oxidative stress ($S_{OS}$) and modified KEAP1 ($K_{1mod}$) from this model, i.e., we do not consider etoposide to directly induce oxidative stress. We have developed three model variants to describe crosstalk upon exposure to etoposide, one for each mode of crosstalk and one for a combination of the two. An overview of the equation numbers forming each model variant and the quality of the simulation fits can be found in Table 1.

#### Crosstalk model DEM

With our second set of crosstalk models, aiming to describe the cellular response to DEM, we focused on the impact of the OSR on the DDR (but not vice versa). We included several modes of crosstalk suggested by literature. First, to simulate DNA damage caused by oxidative stress (due to e.g. reactive oxygen species) [42], we replaced by:


$$\begin{array}{c} \frac{dDD}{dt}=b_{DD}+S_{DD}+\frac{sc_{S}\cdot S_{OX}}{Km_{S}+S_{OX}}-d_{DD,p_{53p}}\cdot p_{53p}\cdot DD\;, \end{array}$$ (16)

in which $sc_{S}$ and $Km_{S}$ are required as additional parameters, respectively representing the maximal rate of DNA damage induction by oxidative stress and the level of oxidative stress for which this rate is half-maximal. Moreover, we set $S_{DD}$ to 0 for all simulations without ’direct’ DNA damage stress.

To model a second mode of crosstalk, i.e., NRF2-dependent MDM2 production [43,44], we replaced by:


$$\begin{array}{c} \frac{dM_{2}}{dt}=b_{M_{2}}+\frac{V_{M_{2}}\cdot p_{53p}^{4}}{Km_{M_{2}}^{4}+p_{53p}^{4}}+\frac{V_{M_{2},N_{2}}\cdot N_{2}^{n_{M_{2}}}}{Km_{M_{2},N_{2}}^{n_{M_{2}}}+N_{2}^{n_{M_{2}}}}-d_{M2}\cdot M_{2}\;, \end{array}$$ (17)

where the new *Km* and *V * parameters (following the prior mentioned naming convention) describe the Hill function leading to additional non-linear MDM2 production.

To model a third mode of crosstalk, i.e., inhibition of basal p53 degradation via the NRF2 target NQO1 [45,54,55], we replaced with:


$$\begin{array}{c} \frac{dp_{53}}{dt}=b_{p_{53}}+r_{dp}\cdot p_{53p}-r_{p}\cdot p_{53}\cdot DD-d_{p_{53}}\cdot \left(\frac{1}{1+C_{S_{1}}\cdot S_{1}}+d_{p_{53},M_{2}}\cdot M_{2}\right)\cdot p_{53}\;. \end{array}$$ (18)

Here the parameter $C_{S_{1}}$ represents the strength of inhibition. Note that we implemented the effect of NQO1 via SRXN1 rather than NQO1. This is because NQO1 is lacking from our OSR model due to absence of measurements of this NRF2 target. HepG2 transcriptomics data shows that NQO1 and SRXN1 are coregulated [57]. Therefore, we expect NQO1 to exhibit similar dynamics as SRXN1, and included it directly as such in our model.

To describe the DEM-induced DDR, we also considered adaptation of the MDM2-dependent p53 degradation from a mass-action term to a non-linear Hill function (with new parameters $Km_{p_{53},M_{2}}$, $Km_{p_{53p},M_{2}}$, $n_{1}$ and $n_{2}$), thus replacing Eqs 4 and 5 with:


$$\begin{array}{c} \frac{dp_{53}}{dt}=b_{p_{53}}+r_{dp}\cdot p_{53p}-r_{p}\cdot p_{53}\cdot DD-d_{p_{53}}\left(1+\frac{d_{p_{53},M_{2}}\cdot M_{2}^{n_{1}}}{Km_{p_{53},M_{2}}^{n_{1}}+M_{2}^{n_{1}}}\right)\cdot p_{53}\;, \end{array}$$ (19)

and:


$$\begin{array}{c} \frac{dp_{53p}}{dt}=r_{p}\cdot p_{53}\cdot DD-r_{dp}\cdot p_{53p}-d_{p_{53p}}\left(1+\frac{d_{p_{53p},M_{2}}\cdot M_{2}^{n_{2}}}{Km_{p_{53p},M_{2}}^{n_{2}}+M_{2}^{n_{2}}}\right)\cdot p_{53p}\;. \end{array}$$ (20)

Combining the adapted MDM2-dependent degradation of p53 and the inhibition of the basal degradation via NQO1 gives the following ODE for unphosphorylated p53:


$$\begin{array}{c} \frac{dp_{53}}{dt}=b_{p_{53}}+r_{dp}\cdot p_{53p}-r_{p}\cdot p_{53}\cdot DD-d_{p_{53}}\left(\frac{1}{1+C_{S_{1}}\cdot S_{1}}+\frac{d_{p_{53},M_{2}}\cdot M_{2}^{n_{1}}}{Km_{p_{53},M_{2}}^{n_{1}}+M_{2}^{n_{1}}}\right)\cdot p_{53}\;. \end{array}$$ (21)

Based on the above 4 changes to the combined model, we implemented six different variants of the crosstalk model upon DEM exposure. In each of the models, we consider direct DNA damage to be absent, i.e. $S_{dd}=0$. An overview of the equation numbers forming each model variants can be found in Table 2.

#### Two-directional crosstalk models

Besides the above one-directional crosstalk models (either crosstalk from OSR to DDR, or vice versa), we also considered two crosstalk models taking into account both directions. First, in model M-CE, we added the crosstalk caused by the activation of the OSR to model M-E1, consisting of Eqs 2–5, 7, 8, 10, 13, 14, and 17. Second, in model M-CD, we added the crosstalk caused by DNA damage to model M-D4, consisting of Eqs 7–11, 13, 14, 16, 17, 19, and 20. As before, for model M-CE, $S_{ox}$ and $K_{1mod}$ was set to 0 and for model M-CD $S_{dd}$ was set to 0. We did not recalibrate or change any of the parameters for these models. To make sure the models would still start at steady state, we first ran them without stress to converge to a steady state and after that applied the stress to the model.

#### Modelling of knockdowns

To simulate knockdown of specific genes, we used models M-CE and M-CD as a basis, and reduced all production terms by 70% in each knockdown simulation. For example, for a knockdown of p53 in model M-CE, this results in:


$$\begin{array}{c} \frac{dp_{53}}{dt}=0.3\cdot \left(b_{p_{53}}\right)+r_{dp}\cdot p_{53p}-r_{p}\cdot p_{53}\cdot DD-d_{p_{53}}\left(1+d_{p_{53},M_{2}}\cdot M_{2}\right)\cdot p_{53}\;, \end{array}$$ (22)

instead of . This way the production rate is reduced to 30% of its normal value without affecting the phosphorylation or degradation of p53. Similarly, knocking down p21 in the same model results in:


$$\begin{array}{c} \frac{dp_{21}}{dt}=0.3\cdot \left(b_{p_{21}}+\frac{V_{p_{21}}\cdot p_{53p}^{4}}{Km_{p_{21}}^{4}+p_{53p}^{4}}\right)-d_{p_{21}}\cdot p_{21}\;, \end{array}$$ (23)

instead of . Here both the basal production and the MDM2-dependent production rates are reduced but the degradation of p21 is unaffected. We performed knockdown simulations in model M-CD in the same manner. Similar to the two-directional crosstalk models, we first let the models converge to a steady state with knockdown but without stress, and next applied stress to the models.

#### Parameter estimation

The parameters of the models were fitted using an in-house fitting script, based on a maximum likelihood approach, previously described in [27,79,87]. In brief, it uses the least-squares method of the SciPy package in Python [88], in combination with sensitivity equations to find the path of the steepest descent towards the optimum [89]. With Latin Hypercube sampling different sets of starting values are generated [90]. The parameter set generating the lowest cost was deemed the optimum.

To ensure finding of the global optimum, a sufficiently large number of initial guesses for parameter sets should be used. For the base models, we used 200 sets, while for the crosstalk models we let the number of sets depend on the number of parameters to be estimated. Specifically, we used 50 sets when calibrating models E1-3 and 100 when fitting models D1-6. Every time we checked if the lowest cost was reached by multiple sets (at least three) to gain confidence that this was indeed the optimum.

We predefined some of the initial values of variables directly based on the data rather than estimating them with parameter calibration because we had direct estimates available based on extrapolation of the data. Specifically, we only estimated the initial values for DNA damage, p53, phosphorylated p53 and unmodified KEAP1 during parameter estimation. For the other, directly measured variables (i.e., MDM2, p21, BTG2, NRF2 and SRXN1), we used the extrapolated value at the 0 hour timepoint as the initial state. The initial value for modified KEAP1 was set to 0.

As an additional constraint on the parameters, we defined steady state constraints in the absence of stress similar to Rosenblatt et al. [91], to ensure the model always starts in a steady state. We achieved this by selecting one parameter from each equation, to be defined by a steady state constraint. These constraints are determined by setting the ODE equal to 0, for a stress with value 0 and the initial state values for the other variables, and then solving this equation for the selected parameter. For equations starting in steady state by definition, i.e. for the stress ($S_{DD}$ and $S_{OS}$) and modified KEAP1 ($K_{1mod}$), no additional constraints were applied to the parameters in those equations.

The lower bound for the fitted parameters was set to 0 and the upper bound to 1000, except for Hill coefficients, which we bounded between 1 and 10. The parameters have arbitrary units because GFP measurements only provide information on changes in relative abundance.

#### Model comparison

Because we created various model variants, we wanted to formally compare how well they described the experimental data. When the number of parameters is the same, one can compare the models by comparing how much each model deviates from the measurements, for example by computing the Residual Squared Sum (RSS)


$$\begin{array}{c} RSS=\overset{N}{\underset{i=1}{\sum }}{\left(y_{i}-f(t_{i})\right)}^{2}\;, \end{array}$$ (24)

with $y_{i}$ the measurement at time point *i*, $f(t_{i})$ the simulation value and *N* the number of time points.

However, when the number of parameters differs, a penalty needs to be included for the number of parameters in order to prevent overfitting. To this end, one can use the Akaike Information Criterion (AIC) [92] or the Bayesian Information Criterion (BIC) [93]. Considering normally distributed residuals, one can use the following expressions to compute the AIC and BIC:


$$\begin{array}{c} AIC=n\cdot \ln\left(\frac{RSS}{n}\right)+2K\;, \end{array}$$ (25)

$$\begin{array}{c} BIC=n\cdot \ln\left(\frac{RSS}{n}\right)+\ln\left(n\right)K\;, \end{array}$$ (26)

with *n* the number of observations, *K* the number of parameters and the *RSS* as defined above. For sample sizes larger than 7, this leads to a larger penalty for an increasing number of parameters in BIC compared to AIC. Because AIC or BIC values do not mean anything on their own, but only the difference in AIC or BIC between two models is relevant, we selected the model with the lowest AIC or BIC as the best model to describe the data.

#### Sensitivity analysis

Once we selected the best model for each drug, we performed a local sensitivity analysis on these models. Specifically, we increased and decreased each parameter separately by 20% and simulated the model for each adapted parameter. Here, we first ran the model without stress to converge to a steady state, and we next ran simulations with stress to the model. To obtain a measure for the effect of changing the parameter, for each model variable the absolute value of its difference between simulations with increased and decreased parameters was summed over all concentrations and time points (evaluated at 1-hour time points; S11A and S12A Figs).

### Used software and data and code availability

For data analysis and data representation, we used R (version 4.3.3) [86] and the tidyverse package (version 2.0.0) [94]. For model simulations, we used the deSolve package (version 1.40) [95] in R. For parameter optimization we used Python (version 3.6.7). Schematic overviews of the pathways were made using Cytoscape [96]. All code to simulate the models and generate the figures is available at doi: 10.5281/zenodo.14844666.

## Supporting information

S1 Fig Viability data for eight concentrations of etoposide. (PDF)

S2 Fig DDR model describes data from HepG2 cells exposed to etoposide. (PDF)

S3 Fig Initial value for the estimated stress levels for each compound concentration. (PDF)

S4 Fig HepG2 cell viability data for eight concentrations of DEM. (PDF)

S5 Fig OSR model describes data from HepG2 cells exposed to DEM when NRF2 stimulates KEAP1 production. (PDF)

S6 Fig Expression of p53 up to 20 hours after exposure to DEM. (PDF)

S7 Fig Experimental data for BTG2 and p21 intensity after exposure of HepG2 cells to eight concentrations of DEM. (PDF)

S8 Fig Crosstalk models to describe etoposide-induced OSR activity. (PDF)

S9 Fig Crosstalk models to describe DEM-induced DDR activity. (PDF)

S10 Fig Crosstalk model to describe DEM-induced DDR activity with altered MDM2 production. (PDF)

S11 Fig Sensitivity analysis for model M-E1. (PDF)

S12 Fig Sensitivity analysis for model M-D4. (PDF)

S13 Fig Best DDR-OSR crosstalk models. (PDF)

S14 Fig *In silico* knockdown predictions on the basis of crosstalk models. (PDF)

S15 Fig *In silico* knockdown predictions for p21. (PDF)

S1 Table Parameter values for the DDR base model. (PDF)

S2 Table Parameter values for the OSR base models with and without NRF2-dependent KEAP1 production. (PDF)

S3 Table New or changed parameters for models M-E1, M-E2 and M-E3. (PDF)

S4 Table New or changed parameters for models M-D1, M-D2 and M-D3. (PDF)

S5 Table New or changed parameters for models M-D4, M-D5 and M-D6. (PDF)
