# Supplementary material for: Computational modelling identifies primary mediators of crosstalk between DNA damage and oxidative stress responses
Source: PLoS Comput Biol. 2025 Mar 10;21(3):e1012844. doi: 10.1371/journal.pcbi.1012844 (PMC12143901; doi:10.1371/journal.pcbi.1012844)
Supplement: S1 Fig — (PDF) [file pcbi.1012844.s001.pdf]

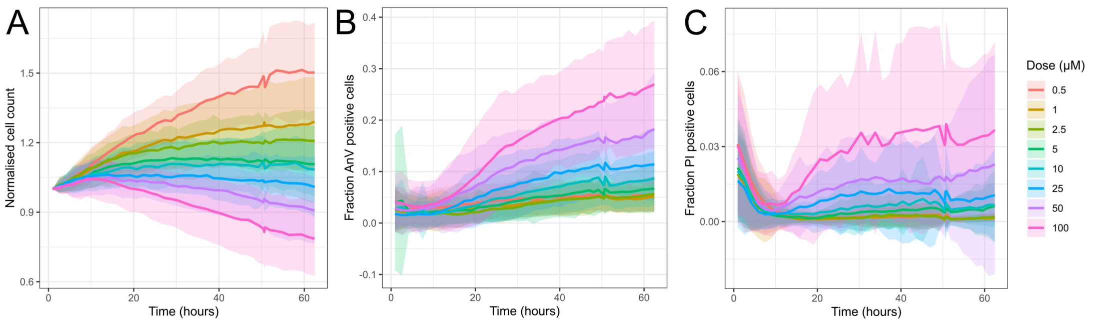

Figure S1: Viability data for eight concentrations of etoposide. A) Normalised cell count, B) fraction AnV-positive cells and C) fraction PI-positive cells. The colour represents the dose and the shaded area indicates the standard deviation across 34 biological replicates (at least three replicates for each of 11 different reporters).
