# Supplementary material for: Computational modelling identifies primary mediators of crosstalk between DNA damage and oxidative stress responses
Source: PLoS Comput Biol. 2025 Mar 10;21(3):e1012844. doi: 10.1371/journal.pcbi.1012844 (PMC12143901; doi:10.1371/journal.pcbi.1012844)
Supplement: S2 Fig — (PDF) [file pcbi.1012844.s002.pdf]

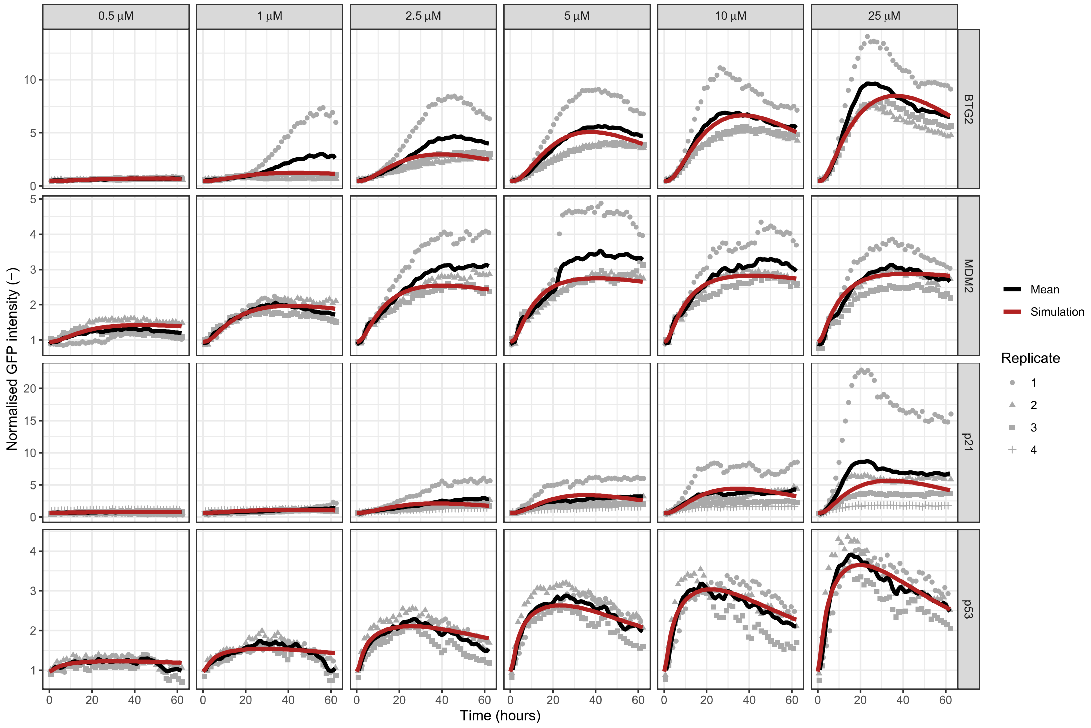

Figure S2: DDR model describes data from HepG2 cells exposed to etoposide. Simulations (red) of the DDR model for BTG2, MDM2, p21 and p53 are shown alongside experimental data (black line represents the mean, grey points the measurements per replicate) for these proteins following exposure to six concentrations of etoposide (in µM).
