# Supplementary material for: Computational modelling identifies primary mediators of crosstalk between DNA damage and oxidative stress responses
Source: PLoS Comput Biol. 2025 Mar 10;21(3):e1012844. doi: 10.1371/journal.pcbi.1012844 (PMC12143901; doi:10.1371/journal.pcbi.1012844)
Supplement: S3 Fig — (PDF) [file pcbi.1012844.s003.pdf]

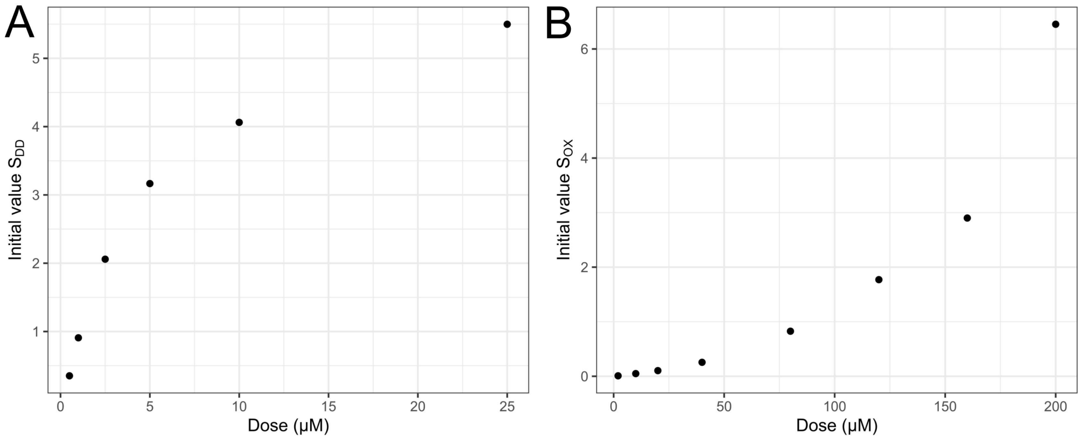

Figure S3: Initial value for the estimated stress levels for each compound concentration. (A-B) Each dot represents the initial value of the stress for each concentration of etoposide (A) or DEM (B) that was used for model simulations.
