# Supplementary material for: Computational modelling identifies primary mediators of crosstalk between DNA damage and oxidative stress responses
Source: PLoS Comput Biol. 2025 Mar 10;21(3):e1012844. doi: 10.1371/journal.pcbi.1012844 (PMC12143901; doi:10.1371/journal.pcbi.1012844)
Supplement: S5 Fig — (PDF) [file pcbi.1012844.s005.pdf]

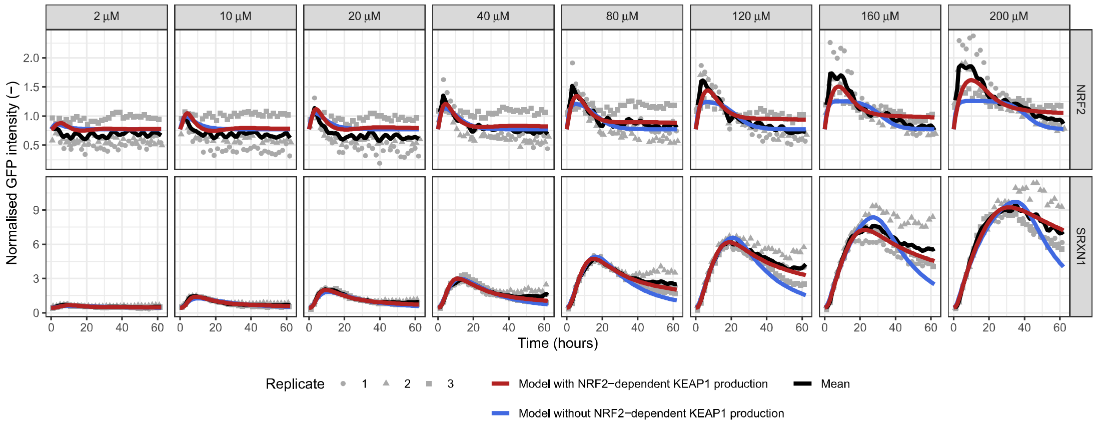

Figure S5: OSR model describes data from HepG2 cells exposed to DEM when NRF2 stimulates KEAP1 production. Simulations of the OSR model with (red) and without (blue) NRF2-dependent KEAP1 production combined with data (black line represents the mean, grey points the measurements per replicate) for these proteins after exposure to eight concentrations of DEM (in µM).
