# Supplementary material for: Computational modelling identifies primary mediators of crosstalk between DNA damage and oxidative stress responses
Source: PLoS Comput Biol. 2025 Mar 10;21(3):e1012844. doi: 10.1371/journal.pcbi.1012844 (PMC12143901; doi:10.1371/journal.pcbi.1012844)
Supplement: S6 Fig — (PDF) [file pcbi.1012844.s006.pdf]

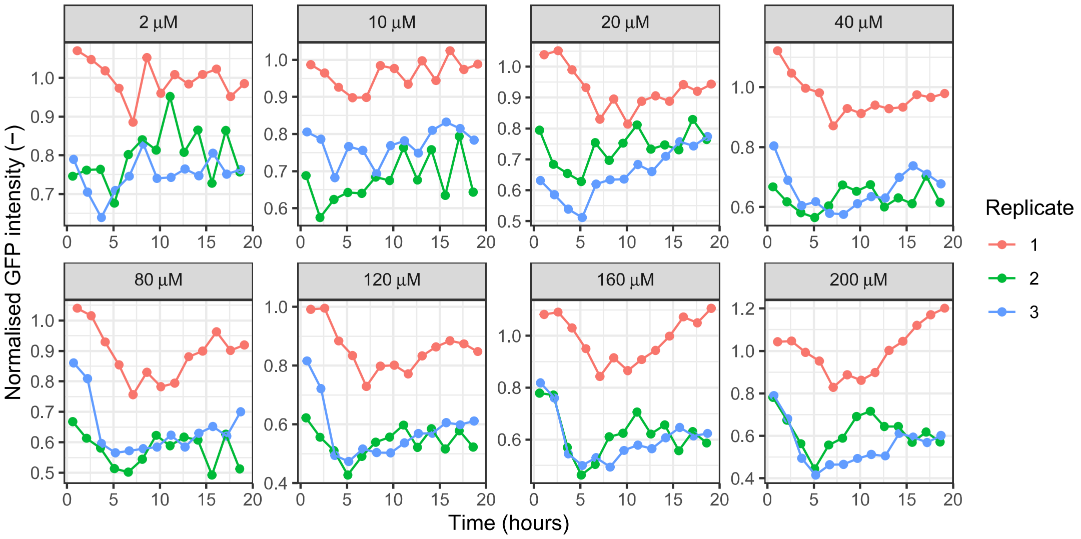

Figure S6: Expression of p53 up to 20 hours after exposure to DEM. Each dot represents an experimental measurement and each colour represents one biological replicate.
