# Supplementary material for: Computational modelling identifies primary mediators of crosstalk between DNA damage and oxidative stress responses
Source: PLoS Comput Biol. 2025 Mar 10;21(3):e1012844. doi: 10.1371/journal.pcbi.1012844 (PMC12143901; doi:10.1371/journal.pcbi.1012844)
Supplement: S7 Fig — (PDF) [file pcbi.1012844.s007.pdf]

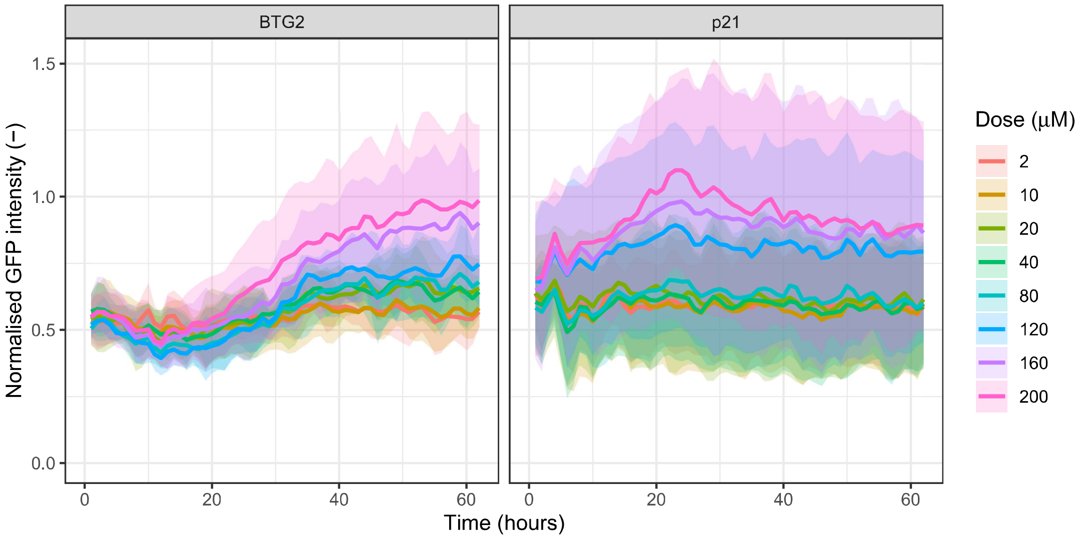

Figure S7: Experimental data for BTG2 and p21 intensity after exposure of HepG2 cells to eight concentrations of DEM. The colour represents the dose and the shaded area indicates the standard deviation across 3 (BTG2) or 4 (p21) biological replicates.
