# Supplementary material for: Computational modelling identifies primary mediators of crosstalk between DNA damage and oxidative stress responses
Source: PLoS Comput Biol. 2025 Mar 10;21(3):e1012844. doi: 10.1371/journal.pcbi.1012844 (PMC12143901; doi:10.1371/journal.pcbi.1012844)
Supplement: S8 Fig — (PDF) [file pcbi.1012844.s008.pdf]

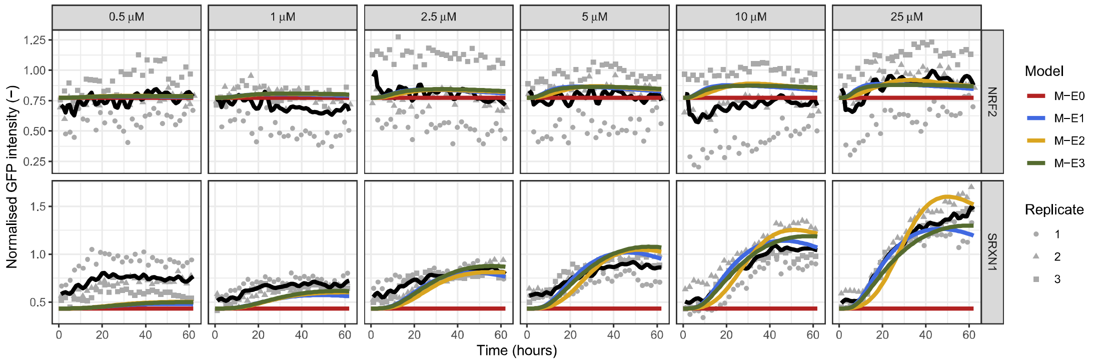

Figure S8: Crosstalk models to describe etoposide-induced OSR activity. Simulations of four different model versions (coloured lines, see Table 1) for NRF2 and SRXN1 are shown alongside experimental data (black line represents the mean, grey points the measurements per replicate) for these proteins after exposure of HepG2 cells to six concentrations of etoposide (in µM).
