# Supplementary material for: Computational modelling identifies primary mediators of crosstalk between DNA damage and oxidative stress responses
Source: PLoS Comput Biol. 2025 Mar 10;21(3):e1012844. doi: 10.1371/journal.pcbi.1012844 (PMC12143901; doi:10.1371/journal.pcbi.1012844)
Supplement: S9 Fig — (PDF) [file pcbi.1012844.s009.pdf]

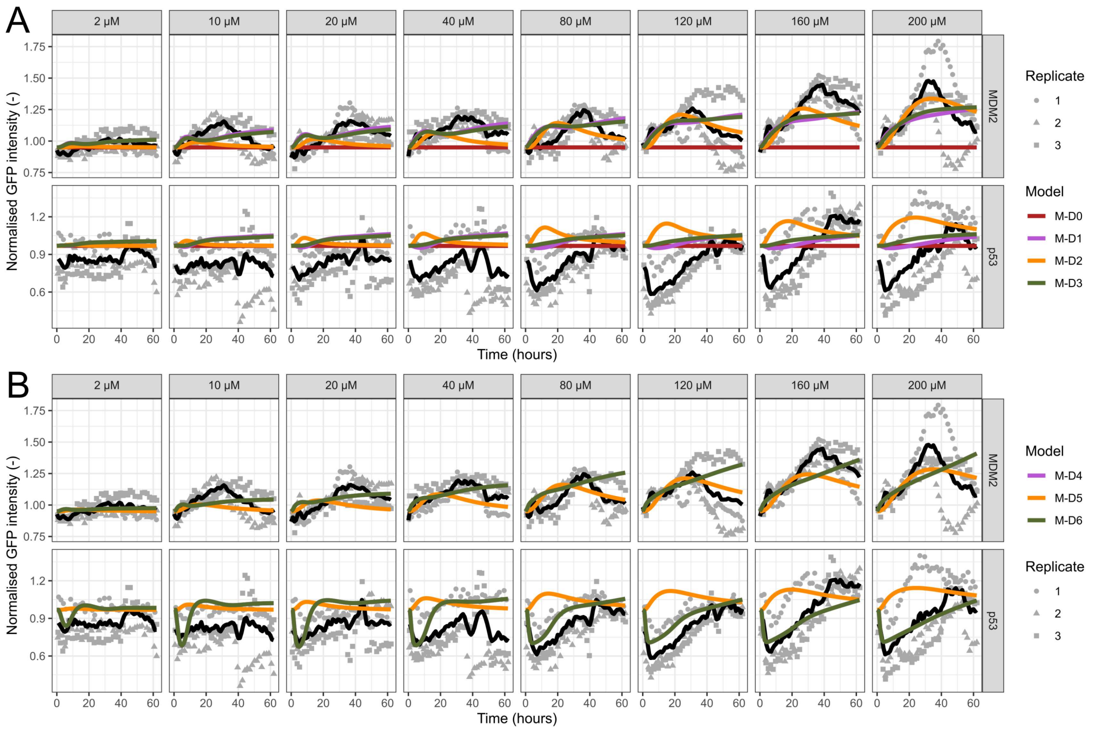

Figure S9: Crosstalk models to describe DEM-induced DDR activity. Simulations of different model versions (coloured lines, see Table 2) for MDM2 and p53 are shown alongside experimental data (black line represents the mean, grey points the measurements per replicate) for these proteins after exposure of HepG2 cells to eight concentrations of etoposide (in μM). Models without (A) and with (B) adapted MDM2-dependent p53 degradation.
