# Supplementary material for: Computational modelling identifies primary mediators of crosstalk between DNA damage and oxidative stress responses
Source: PLoS Comput Biol. 2025 Mar 10;21(3):e1012844. doi: 10.1371/journal.pcbi.1012844 (PMC12143901; doi:10.1371/journal.pcbi.1012844)
Supplement: S10 Fig — (PDF) [file pcbi.1012844.s010.pdf]

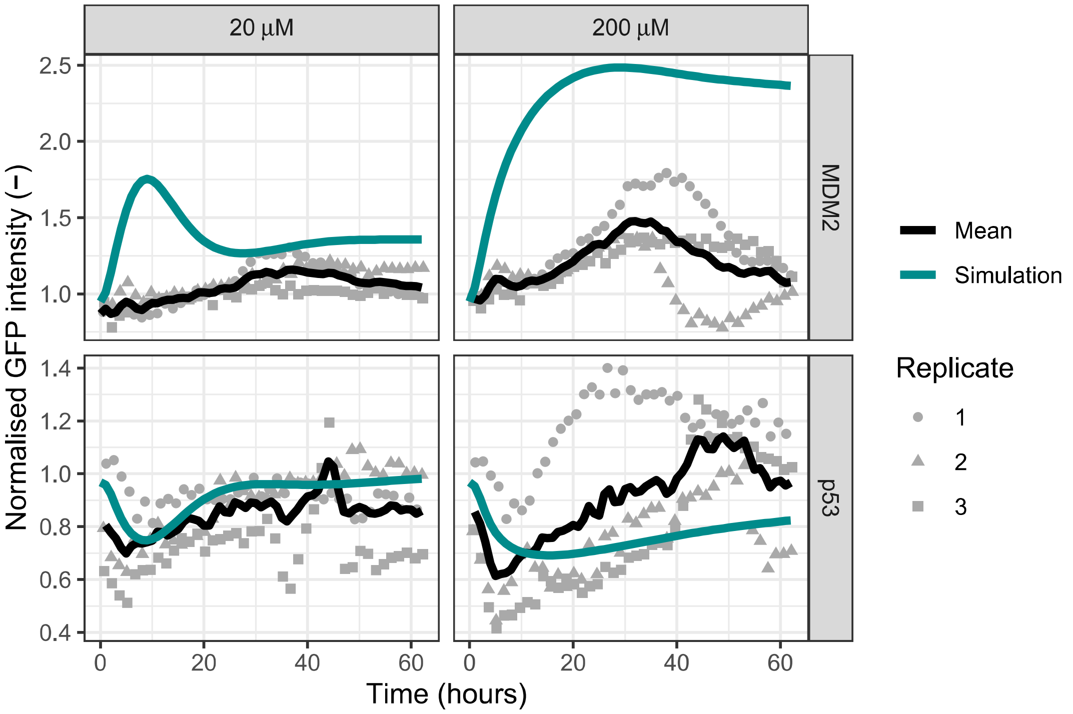

Figure S10: Crosstalk model to describe DEM-induced DDR activity with altered MDM2 production. Model M-D3 was adapted to have an 8-fold higher maximal NRF2-dependent MDM2 production rate compared to the base model M-D3. Model simulations (blue) for MDM2 and p53 are shown alongside experimental data (black line represents the mean, grey points the measurements per replicate) for these proteins after exposure of HepG2 cells to 20 and 200 µM DEM.
