# Supplementary material for: Computational modelling identifies primary mediators of crosstalk between DNA damage and oxidative stress responses
Source: PLoS Comput Biol. 2025 Mar 10;21(3):e1012844. doi: 10.1371/journal.pcbi.1012844 (PMC12143901; doi:10.1371/journal.pcbi.1012844)
Supplement: S12 Fig — (PDF) [file pcbi.1012844.s012.pdf]

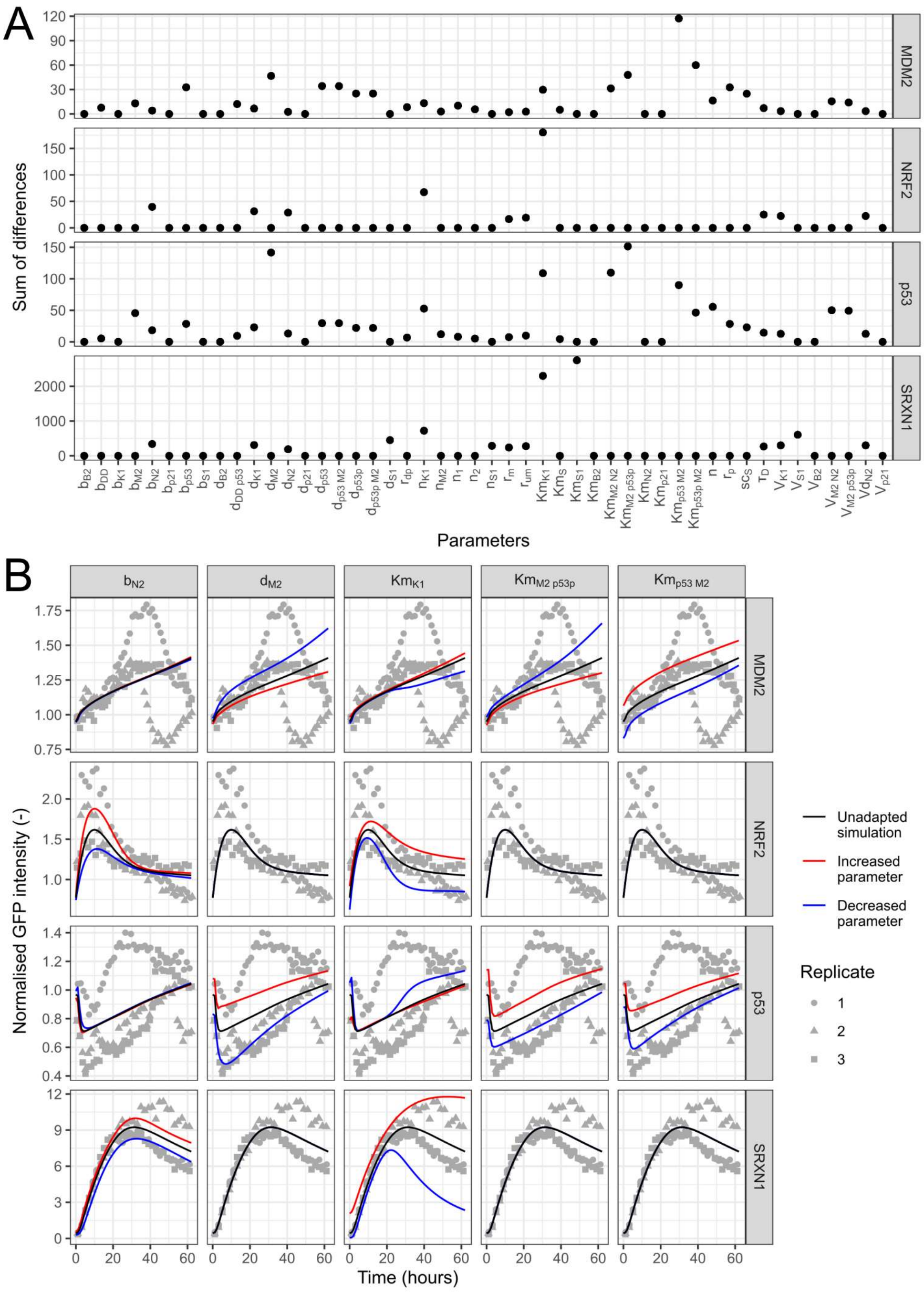

Figure S12: Sensitivity analysis for model M-D4. A) The effect of varying each model parameter on the four main system variables for model M-D4. Per variable the absolute difference in the simulation output for each increased and decreased parameter was summed over all time points and concentrations. Each dot represents the sum of these differences for one parameter and variable. B) Comparison of simulations for exposure to 200 µM DEM (lines) and experimental data (symbols). Results are shown for the simulation without any parameter adaptations (black), the simulation with specific parameters (indicated on top of each column) increased (red) or decreased (blue) by 20 %.
