# Supplementary material for: Computational modelling identifies primary mediators of crosstalk between DNA damage and oxidative stress responses
Source: PLoS Comput Biol. 2025 Mar 10;21(3):e1012844. doi: 10.1371/journal.pcbi.1012844 (PMC12143901; doi:10.1371/journal.pcbi.1012844)
Supplement: S13 Fig — (PDF) [file pcbi.1012844.s013.pdf]

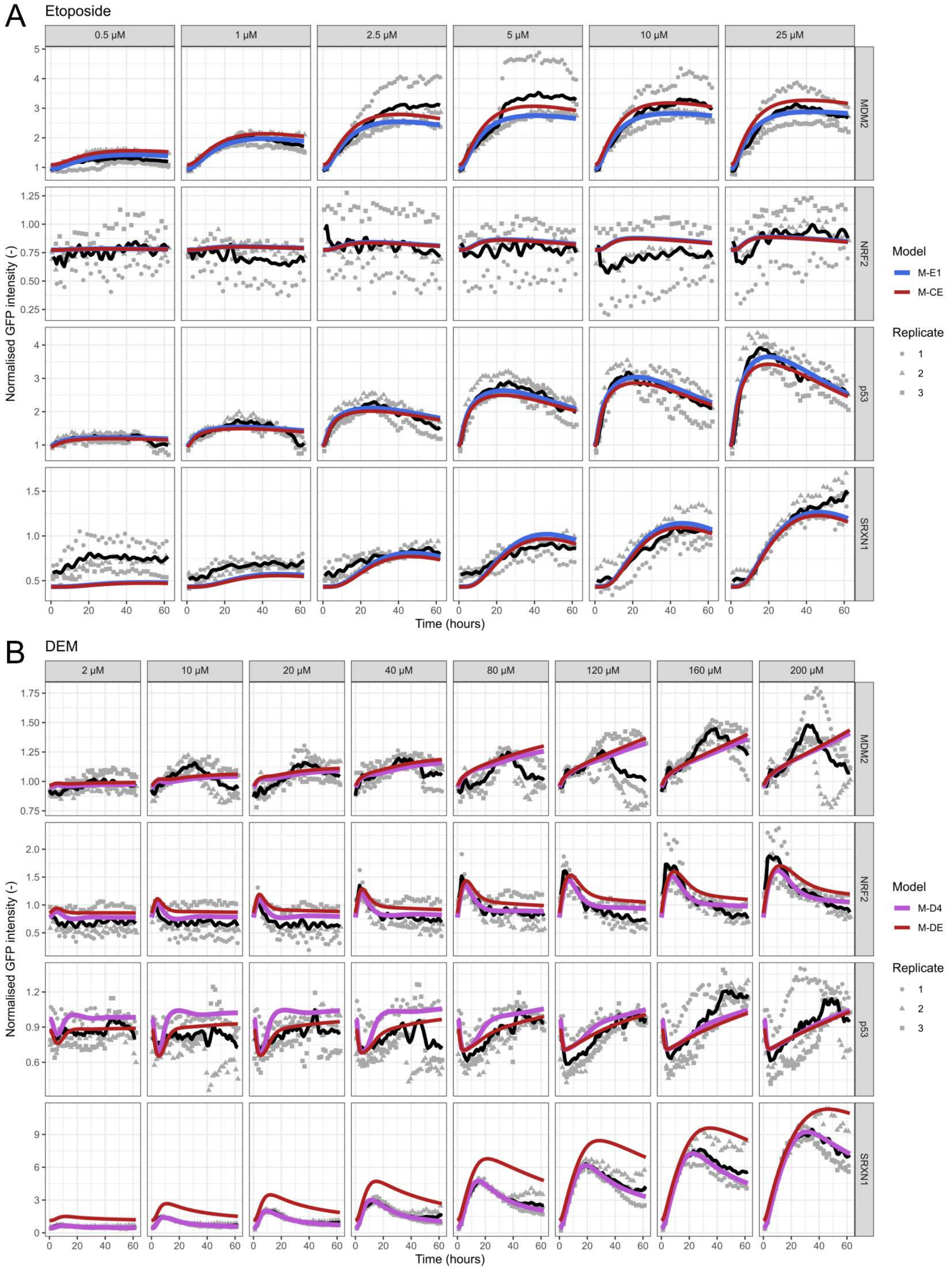

Figure S13: Best DDR-OSR crosstalk models. A-B) Simulations of best unidirectional crosstalk model versions (blue or pink) and two-directional crosstalk models (red) for exposure to etoposide (A) or DEM (B). Simulations (coloured lines) and experimental data (black line represents the mean, grey points the measurements per replicate) are shown for MDM2, NRF2, p53 and SRXN1 after exposure of HepG2 cells to the indicated compounds.
