# Supplementary material for: Computational modelling identifies primary mediators of crosstalk between DNA damage and oxidative stress responses
Source: PLoS Comput Biol. 2025 Mar 10;21(3):e1012844. doi: 10.1371/journal.pcbi.1012844 (PMC12143901; doi:10.1371/journal.pcbi.1012844)
Supplement: S14 Fig — (PDF) [file pcbi.1012844.s014.pdf]

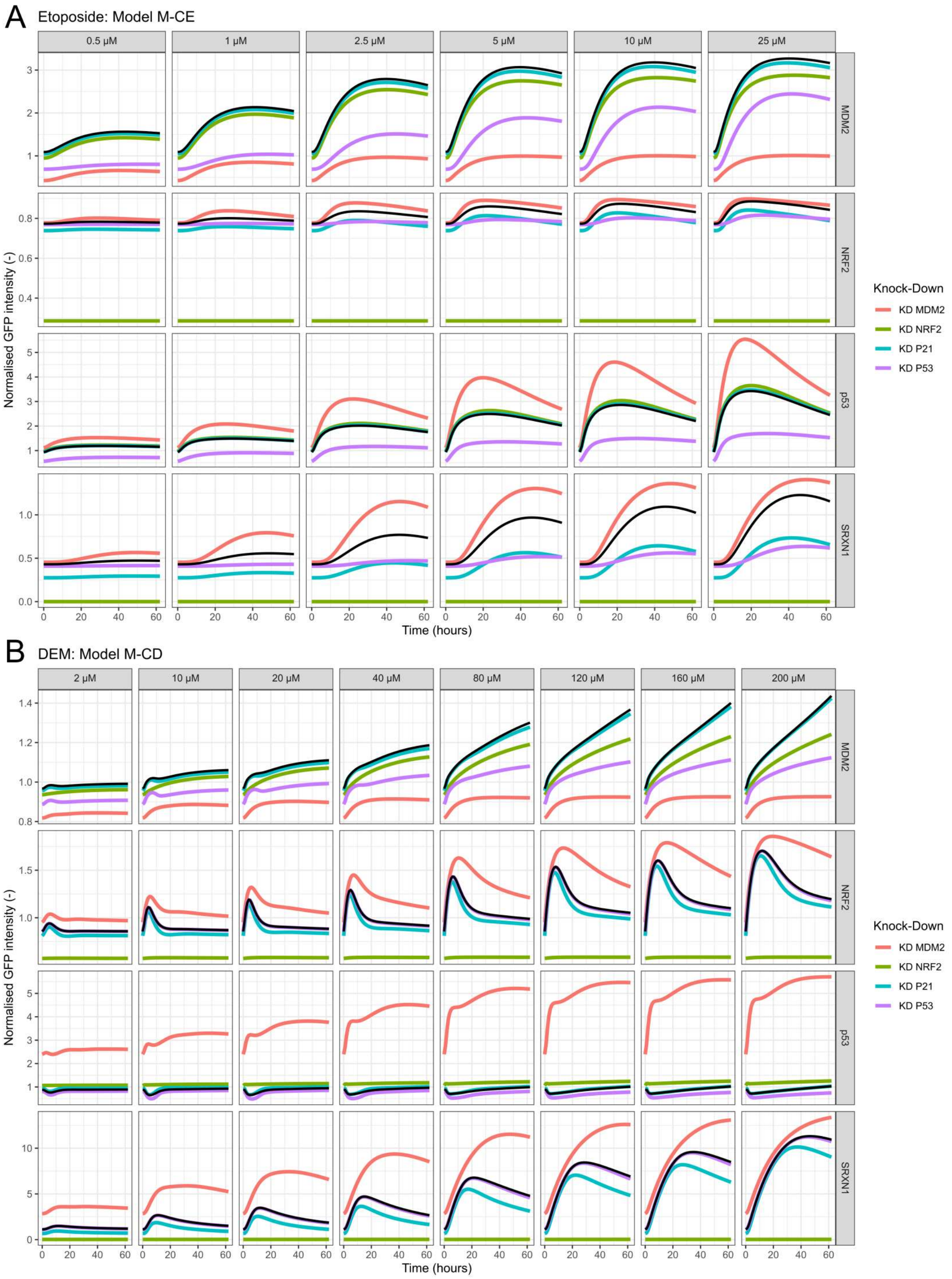

Figure S14: *In silico* knockdown predictions on the basis of crosstalk models. A-B) Simulation of knockdown models (coloured lines) and combined models without knockdown (black line) are shown for MDM2, NRF2, p53 and SRXN1, following exposure of HepG2 cells to etoposide (A) or DEM (B).
