# Supplementary material for: Computational modelling identifies primary mediators of crosstalk between DNA damage and oxidative stress responses
Source: PLoS Comput Biol. 2025 Mar 10;21(3):e1012844. doi: 10.1371/journal.pcbi.1012844 (PMC12143901; doi:10.1371/journal.pcbi.1012844)
Supplement: S15 Fig — (PDF) [file pcbi.1012844.s015.pdf]

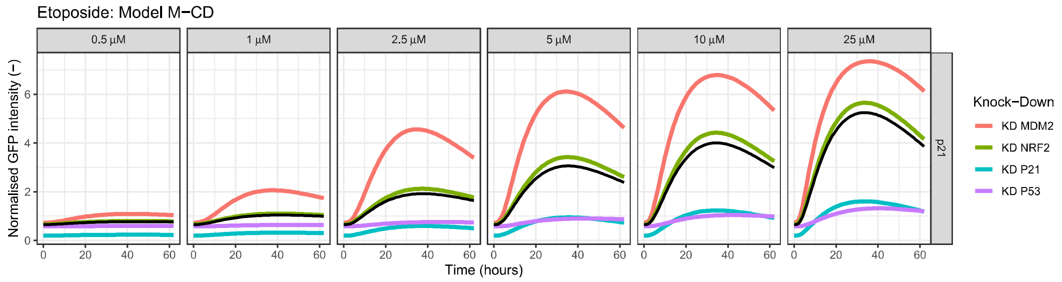

Figure S15: *In silico* knockdown predictions for p21. Simulation of knockdown models (coloured lines) and combined model without knockdown (black line) are shown for p21, following exposure of HepG2 cells to etoposide.
