# Supplementary material for: Computational modelling identifies primary mediators of crosstalk between DNA damage and oxidative stress responses
Source: PLoS Comput Biol. 2025 Mar 10;21(3):e1012844. doi: 10.1371/journal.pcbi.1012844 (PMC12143901; doi:10.1371/journal.pcbi.1012844)
Supplement: S1 Table — (PDF) [file pcbi.1012844.s016.pdf]

Table S1: Parameter values for the DDR base model. The asterisk ($*$) indicates fixed parameter values and the bullet ($\bullet$) indicates values that were computed with the steady state constraints.

**Parameter**	**Unit**	**Description**	**Value**
DD_{ss}	-	Steady-state value for DNA damage	8.571625056
p_{53ss}	au	Steady-state value for p53	0.000180765
p_{53Pss}	au	Steady-state value for phosphorylated p53	0.967836355
M_{2ss}	au	Steady-state value for MDM2	0.9496844 $*$
p_{21ss}	au	Steady-state value for p21	0.659682552 $*$
B_{2ss}	au	Steady-state value for BTG2	0.455598913 $*$
$EC1$	-	Initial stress value for 0.5 µM etoposide	0.351503219
$EC2$	-	Initial stress value for 1 µM etoposide	0.908163878
$EC3$	-	Initial stress value for 2.5 µM etoposide	2.058779232
$EC4$	-	Initial stress value for 5 µM etoposide	3.165986656
$EC5$	-	Initial stress value for 10 µM etoposide	4.061987716
$EC6$	-	Initial stress value for 25 µM etoposide	5.498461789
τ_D	hr^{-1}	Stress decay rate	0.040028179
b_{DD}	hr^{-1}	Basal DNA damage production rate	0.082208747
$b_{p_{53}}$	au/hr	Basal p53 production rate	999.9948663
r_{dp}	hr^{-1}	Dephosphorylation rate p53p	0.728189372
r_p	hr^{-1}	Phosphorylation rate of p53	999.9969129
d_{p_{53},M_2}	$au^{-1}hr^{-1}$	MDM2-mediated p53 degradation rate	3.22E-07
d_{p_{53p},M_2}	$au^{-1}hr^{-1}$	MDM2-mediated p53p degradation rate	4.641296207
b_{M_2}	au/hr	Basal MDM2 production rate	0.048913592
V_{M_2}	au/hr	Maximal p53-dependent MDM2 production rate	0.287645017
Km_{M_2}	au	Michaelis-Menten constant for MDM2 production	1.359464444
$b_{p_{21}}$	au/hr	Basal p21 production rate	0.058365284
V_{p_21}	au/hr	Maximal p53-dependent p21 production rate	0.80903637
$Km_{p_{21}}$	au	Michaelis-Menten constant for p21 production	2.915716363
b_{B_2}	au/hr	Basal BTG2 production rate	0.024200176
V_{B_2}	au/hr	Maximal p53-dependent BTG2 production rate	1.061170467
Km_{B_2}	au	Michaelis-Menten constant for BTG2 production	2.808959435
$d_{DD,p_{53p}}$	$au^{-1}hr^{-1}$	p53-dependent DNA damage degradation rate	0.009909527 $\bullet$
$d_{p_{53}}$	hr^{-1}	Basal p53 degradation rate	5527345.385 $\bullet$
d_{P53p}	hr^{-1}	Basal phosphorylated p53 degradation rate	0.161387603 $\bullet$
d_{M2}	hr^{-1}	Basal MDM2 degradation rate	0.113409117 $\bullet$
$d_{p_{21}}$	hr^{-1}	Basal p21 degradation rate	0.103185017 $\bullet$
d_{B_2}	hr^{-1}	Basal BTG2 degradation rate	0.085487843 $\bullet$
