# Supplementary material for: Computational modelling identifies primary mediators of crosstalk between DNA damage and oxidative stress responses
Source: PLoS Comput Biol. 2025 Mar 10;21(3):e1012844. doi: 10.1371/journal.pcbi.1012844 (PMC12143901; doi:10.1371/journal.pcbi.1012844)
Supplement: S2 Table — (PDF) [file pcbi.1012844.s017.pdf]

Table S2: Parameter values for the OSR base models with and without NRF2-dependent KEAP1 production. The asterisk ($*$) indicates fixed parameter values and the bullet ($\bullet$) indicates values that were computed with the steady state constraints.

**Parameter**	**Unit**	**Description**	**With NRF2-dependent KEAP1 production**	**Without NRF2-dependent KEAP1 production**
K_{1ss}	au	Steady-state value KEAP1	83.82597611	1.518996292
K_{1modss}	au	Steady-state value modified KEAP1	0$*$	0$*$
N_{2ss}	au	Steady-state value NRF2	0.77378141 $*$	0.77378141 $*$
S_{1ss}	au	Steady state value SRXN1	0.433384695 $*$	0.433384695 $*$
$EC1$	-	Initial stress value for 2 µM DEM	0.007973741	0.000668075
$EC2$	-	Initial stress value for 10 µM DEM	0.048510948	0.002660588
$EC3$	-	Initial stress value for 20 µM DEM	0.103026076	0.004591022
$EC4$	-	Initial stress value for 40 µM DEM	0.255548911	0.010729464
$EC5$	-	Initial stress value for 80 µM DEM	0.825573528	0.041050546
$EC6$	-	Initial stress value for 120 µM DEM	1.770714715	0.156556903
$EC7$	-	Initial stress value for 160 µM DEM	2.900508362	0.978487052
$EC8$	-	Initial stress value for 200 µM DEM	6.452682665	6.907755278
τ_O	hr^{-1}	Stress decay rate	0.018758805	0.210351105
b_{K_1}	au/hr	Basal KEAP1 production rate	3.682008930e-12	0.053308531
r_m	hr^{-1}	KEAP1 modification rate rate	29.83805612	999.9999995
r_{um}	hr^{-1}	Modified KEAP1 unmodification rate	0.724483506	1.208493854
V_{K_1}	au/hr	Maximal NRF2-dependent KEAP1 production rate	989.0717892	-
Km_{K_1}	au	Michaelis-Menten constant for KEAP1 production	1.434512426	-
n_{K_1}	-	Hill-coefficient KEAP1 production	9.999999998	-
b_{N_2}	au/hr	Basal NRF2 production	0.364673872	0.946100057
Vd_{N_2}	$au^{-1}hr^{-1}$	Maximal rate of NRF2 degradation	0.002626465	0.952948605
Km_{N_2}	au	Michaelis-Menten constant for NRF2 degradation	1.554999290e-13	2.292462387
b_{S_1}	au/hr	Basal SRXN1 production rate	7.479035267e-16	2.44E-07
V_{S_1}	au/hr	Maximal NRF2-dependent SRXN1 production rate	0.666168660	0.784698782
Km_{S_1}	au	Michaelis-Menten constant for SRXN1 production	1.093301340	1.110862324
n_{S_1}	-	Hill coefficient SRXN1 production	9.999999999	9.999999999
d_{K_1}	hr^{-1}	Basal (modified) KEAP1 degradation rate	0.024551856 $\bullet$	0.035094576 $\bullet$
d_{N_2}	hr^{-1}	Basal NRF2 degradation rate	0.186755404 $\bullet$	0.750612557 $\bullet$
d_{S_1}	hr^{-1}	Basal SRXN1 degradation rate	0.046990605 $\bullet$	0.047412347 $\bullet$
