# Supplementary material for: Computational modelling identifies primary mediators of crosstalk between DNA damage and oxidative stress responses
Source: PLoS Comput Biol. 2025 Mar 10;21(3):e1012844. doi: 10.1371/journal.pcbi.1012844 (PMC12143901; doi:10.1371/journal.pcbi.1012844)
Supplement: S3 Table — (PDF) [file pcbi.1012844.s018.pdf]

Table S3: New or changed parameters for models M-E1, M-E2 and M-E3. The bullet (•) indicates values that were computed with the steady state constraints.

**Model**	**Parameter**	**Unit**	**Description**	**Value**
M-E1	$C_{p_{21}}$	-	Crosstalk parameter p21-NRF2	2.606223583
M-E1	d_{N_2}	hr^{-1}	Basal NRF2 degradation rate	0.382976513 •
M-E2	$C_{p_{53p}}$	-	Crosstalk parameter p53p-KEAP1	0.116291804
M-E2	d_{K_1}	hr^{-1}	Basal NRF2 degradation rate	0.0115485 •
M-E3	$C_{p_{21}}$	-	Crosstalk parameter p21-NRF2	3.741425878
M-E3	$C_{p_{53p}}$	-	Crosstalk parameter p53p-KEAP1	0.054718344
M-E3	d_{K_1}	hr^{-1}	Basal KEAP1 degradation rate	0.017096964 •
M-E3	d_{N_2}	hr^{-1}	Basal NRF2 degradation rate	0.403376093 •
