# Supplementary material for: Computational modelling identifies primary mediators of crosstalk between DNA damage and oxidative stress responses
Source: PLoS Comput Biol. 2025 Mar 10;21(3):e1012844. doi: 10.1371/journal.pcbi.1012844 (PMC12143901; doi:10.1371/journal.pcbi.1012844)
Supplement: S4 Table — (PDF) [file pcbi.1012844.s019.pdf]

Table S4: New or changed parameters for models M-D1, M-D2 and M-D3. The bullet (•) indicates values that were computed with the steady state constraints.

**Model**	**Parameter**	**Unit**	**Description**	**Value**
M-D1	sc_S	-	Scale parameter for ROS-induced DNA damage	0.045883095
M-D1	Km_S	-	Michaelis-Menten constant oxidative stress	0.008932081
M-D1	V_{M_2,N_2}	au/hr	Maximal NRF2-dependent MDM2 production rate	0.036445016
M-D1	n_{M_2}	-	Hill coefficient MDM2 production	10
M-D1	Km_{M_2,N_2}	au	Michaelis-Menten constant for NRF2-dependent MDM2 production	0.913496247
M-D1	d_{M_2}	hr^{-1}	Basal MDM2 degradation rate	0.119540722 •
M-D2	C_{S_1}	au^{-1}	Crosstalk parameter SRXN1-p53	0.059160628
M-D2	V_{M_2,N_2}	au/hr	Maximal NRF2-dependent MDM2 production rate	1.03E-27
M-D2	n_{M_2}	-	Hill coefficient MDM2 production	9.997951894
M-D2	Km_{M_2,N_2}	au	Michaelis-Menten constant for NRF2-dependent MDM2 production	992.1946845
M-D2	d_{p53}	hr^{-1}	Basal p53 degradation rate	5669062.001 •
M-D2	d_{M_2}	hr^{-1}	Basal MDM2 degradation rate	0.113409185 •
M-D3	sc_S	-	Scale parameter for ROS-induced DNA damage	0.037830853
M-D3	Km_S	-	Michaelis-Menten constant oxidative stress	0.006621225
M-D3	C_{S_1}	au^{-1}	Crosstalk parameter SRXN1-p53	0.011113252
M-D3	V_{M_2,N_2}	au/hr	Maximal NRF2-dependent MDM2 production rate	0.029943324
M-D3	n_{M_2}	-	Hill coefficient MDM2 production	9.999988682
M-D3	Km_{M_2,N_2}	au	Michaelis-Menten constant for NRF2-dependent MDM2 production	0.906347196
M-D3	d_{p53}	hr^{-1}	Basal p53 degradation rate	5553966.152 •
M-D3	d_{M_2}	hr^{-1}	Basal MDM2 degradation rate	0.118788396 •
