# Supplementary material for: Computational modelling identifies primary mediators of crosstalk between DNA damage and oxidative stress responses
Source: PLoS Comput Biol. 2025 Mar 10;21(3):e1012844. doi: 10.1371/journal.pcbi.1012844 (PMC12143901; doi:10.1371/journal.pcbi.1012844)
Supplement: S5 Table — (PDF) [file pcbi.1012844.s020.pdf]

Table S5: New or changed parameters for models M-D4, M-D5 and M-D6. The bullet (•) indicates values that were computed with the steady state constraints.

Model	Parameter	Unit	Description	Value
M-D4	sc_S	-	Scale parameter for ROS-induced DNA damage	2.145438657
M-D4	Km_S	-	Michaelis-Menten constant oxidative stress	0.127521435
M-D4	V_{M_2,N_2}	au/hr	Maximal NRF2-dependent MDM2 production rate	0.085530357
M-D4	n_{M_2}	-	Hill coefficient MDM2 production	9.999999998
M-D4	Km_{M_2,N_2}	au	Michaelis-Menten constant for NRF2-dependent MDM2 production	0.861005574
M-D4	d_{p_{53},M_2}	hr^{-1}	Maximal increase in MDM2-dependent p53 degradation	998.74198
M-D4	n_1	-	Hill coefficient p53 degradation	9.999999959
M-D4	Km_{p_{53},M_2}	au	Michaelis-Menten constant for p53	1.101669604
M-D4	d_{p_{53p},M_2}	hr^{-1}	Maximal increase in MDM2-dependent p53p degradation	999.8961236
M-D4	n_2	-	Hill coefficient p53p degradation	9.999999991
M-D4	Km_{p_{53p},M_2}	au	Michaelis-Menten constant for p53p	1.043643213
M-D4	$d_{p_{53}}$	hr^{-1}	Basal p53 degradation rate	29794.98567 •
M-D4	$d_{p_{53p}}$	hr^{-1}	Basal phosphorylated p53 degradation rate	0.003103889 •
M-D4	d_{M_2}	hr^{-1}	Basal MDM2 degradation rate	0.136443555 •
M-D5	C_{S_1}	au^{-1}	Crosstalk parameter SRXN1-p53	0.201013302
M-D5	V_{M_2,N_2}	au/hr	Maximal NRF2-dependent MDM2 production rate	0.005399069
M-D5	n_{M_2}	-	Hill coefficient MDM2 production	9.999991673
M-D5	Km_{M_2,N_2}	au	Michaelis-Menten constant for NRF2-dependent MDM2 production	0.905785494
M-D5	d_{p_{53},M_2}	hr^{-1}	Maximal increase in MDM2-dependent p53 degradation	2.272749409
M-D5	n_1	-	Hill coefficient p53 degradation	1.006476862
M-D5	Km_{p_{53},M_2}	au	Michaelis-Menten constant for p53	6.01E-95
M-D5	d_{p_{53p},M_2}	hr^{-1}	Maximal increase in MDM2-dependent p53p degradation	4.64E-14
M-D5	n_2	-	Hill coefficient p53p degradation	9.996821898
M-D5	Km_{p_{53p},M_2}	au	Michaelis-Menten constant for p53p	980.9953743
M-D5	$d_{p_{53}}$	hr^{-1}	Basal p53 degradation rate	1731291.595 •
M-D5	$d_{p_{53p}}$	hr^{-1}	Basal phosphorylated p53 degradation rate	0.872746598 •
M-D5	d_{M_2}	hr^{-1}	Basal MDM2 degradation rate	0.114384105 •
M-D6	C_{S_1}	au^{-1}	Crosstalk parameter SRXN1-p53	6.31E-06
M-D6	V_{M_2,N_2}	au/hr	Maximal NRF2-dependent MDM2 production rate	0.08536444
M-D6	n_{M_2}	-	Hill coefficient MDM2 production	9.999999998
M-D6	Km_{M_2,N_2}	au	Michaelis-Menten constant for NRF2-dependent MDM2 production	0.861493523
M-D6	sc_S	-	Scale parameter for ROS-induced DNA damage	2.099063553
M-D6	Km_S	-	Michaelis-Menten constant oxidative stress	0.126657434
M-D6	d_{p_{53},M_2}	hr^{-1}	Parameter for MDM2-dependent p53 degradation	243.6909724
M-D6	n_1	-	Hill coefficient p53 degradation	9.999964497
M-D6	Km_{p_{53},M_2}	au	Michaelis-Menten constant for p53	1.100871694
M-D6	d_{p_{53p},M_2}	hr^{-1}	Maximal increase in MDM2-dependent p53p degradation	177.3207948
M-D6	n_2	-	Hill coefficient p53p degradation	9.999973846
M-D6	Km_{p_{53p},M_2}	au	Michaelis-Menten constant for p53p	1.045312926
M-D6	$d_{p_{53}}$	hr^{-1}	Basal p53 degradation rate	119413.5556 •
M-D6	$d_{p_{53p}}$	hr^{-1}	Basal phosphorylated p53 degradation rate	0.017414184 •
M-D6	d_{M_2}	hr^{-1}	Basal MDM2 degradation rate	0.136302068 •
